# Fertility Outcomes in Leukemia Survivors by Treatment Modality: A Systematic Review and Meta-Analysis

**DOI:** 10.3390/cancers18152455

**Published:** 2026-07-30

**Authors:** Judith Altmann, Nadine Einsiedel, Janna Pape, Susanna Weidlinger, Tanya Karrer, Michael von Wolff, Magdalena Balcerek

**Affiliations:** 1 Department of Gynecology with Center of Oncological Surgery, Charité—Universitätsmedizin Berlin, Augusteburger Platz 1, 13353 Berlin, Germany; nadine.einsiedel@charite.de; 2 Division of Gynecological Endocrinology and Reproductive Medicine, University Women’s Hospital, Inselspital Bern, University of Bern, 3010 Bern, Switzerland; janna.pape@insel.ch (J.P.); susanna.weidlinger@insel.ch (S.W.); michael.vonwolff@insel.ch (M.v.W.); 3 Medical Library, University Library Bern, University of Bern, 3012 Bern, Switzerland; tanya.karrer@unibe.ch; 4 Department of Oncology, Hepatology and Pneumology, University Cancer Center Leipzig, University Hospital Leipzig, Liebigstraße 22, 04103 Leipzig, Germany; magdalena.balcerek@medizin.uni-leipzig.de; 5 Department of Pediatric Oncology and Hematology, Charité—Universitätsmedizin Berlin, Augustenburger Platz 1, 13353 Berlin, Germany

**Keywords:** leukemia, gonadotoxicity, oncofertility, fertility preservation, survivorship, FertiTOX, FertiPROTEKT

## Abstract

**Introduction**: Leukemia is the most common childhood malignancy. Treatment requires urgent polychemotherapy and may escalate to hematopoietic stem cell transplantation (HSCT) with or without total body irradiation (TBI). Due to the frequently young age at diagnosis, time limitations and unpredictable course, fertility preservation (FP) is especially challenging and may not be feasible prior to treatment, particularly in females. **Methods**: We conducted a systematic review and meta-analysis of fertility outcomes in leukemia survivors using MEDLINE, Embase, and Cochrane databases (search date: 13 June 2024). **Results**: Forty-two studies with 2048 patients were included. Overall infertility was 37% (95% CI 23–54%) with substantial heterogeneity. The highest infertility rates were observed after HSCT (68%) and after testicular irradiation (92%) or TBI ≥ 10–12 Gy (66%). Chemotherapy alone was associated with lower infertility (4%). Sex-specific differences were noted, with higher infertility in males following chemotherapy-only regimens (18% vs. 6% in females), while females were more affected after HSCT (70% vs. 59% in males). Subfertility affected about one-fifth of survivors. **Conclusions**: While standard chemotherapy is associated with relatively low gonadotoxicity, HSCT and irradiation confer substantial risk. These findings support individualized fertility counseling and long-term reproductive follow-up based on treatment exposure.

## Simple Summary

Childhood leukemia is the most common cancer in children, and advances in treatment have greatly improved survival. As more patients reach adulthood, preserving fertility has become an increasingly important aspect of survivorship care. However, the risk of infertility differs considerably between treatment types, making fertility counseling and decisions about fertility preservation challenging. In this systematic review and meta-analysis, we summarized the available evidence on fertility outcomes after treatment for childhood leukemia. We found that standard chemotherapy alone was associated with a relatively low risk of infertility, whereas stem cell transplantation and radiation therapy were associated with a substantially higher risk. These findings provide a comprehensive overview of treatment-related fertility risks and may help healthcare professionals improve fertility counseling, guide fertility preservation strategies, and optimize long-term follow-up for survivors of childhood leukemia.

## 1. Introduction

Leukemia represents the most common malignant disease in childhood, accounting for almost one third of all cancer diagnoses before the age of 18 years, with acute lymphoblastic leukemia (ALL) being the predominant subtype [1]. In adults, leukemia incidence increases steadily with age, while chronic leukemias remain rare in the pediatric population [2]. The diagnosis of acute leukemia requires immediate initiation of oncologic therapy according to standardized, risk-adapted treatment protocols. Standard therapy consists primarily of polychemotherapy, while additional modalities—such as cranial irradiation (CRT, for central nervous system prophylaxis or involvement), testicular irradiation (for testicular disease or relapse), or allogeneic hematopoietic stem cell transplantation (HSCT)—may be required in selected cases. Approximately 5% of pediatric and up to 50% of adult ALL patients require high-risk treatment with allogeneic HSCT [3,4]. Currently, more than 90% of children with ALL and approximately 76% with acute myeloid leukemia (AML) achieve long-term survival beyond 15 years [1]. In adults, survival rates have also significantly improved and nowadays range between 70 and 80% [2].

With improving survival, the quality of life of former leukemia patients has become a central focus of care. A key life goal associated with high quality of life in adult survivors is the ability to establish a family of their own [5]. However, for cancer patients, treatment-related gonadotoxicity represents a major concern in both sexes. While standard chemotherapy regimens carry a low infertility risk, intensive protocols, particularly conditioning regimens containing alkylating agents and total body irradiation (TBI; typically 12–18 Gy) prior to HSCT, are associated with infertility rates above 80% [6]. Following allogeneic HSCT, infertility may occur immediately or develop gradually, with a median onset of 2.5 years after transplantation [7,8]. Data on the gonadotoxic potential of novel targeted and immune-based therapies remain limited [9]. Given the life-threatening nature of leukemia, the often limited FP options at diagnosis, and the unpredictable need for treatment escalation, more precise prediction of individual gonadotoxic risk is urgently needed.

To date, no comprehensive synthesis of fertility outcomes across leukemia subtypes and treatment strategies exists in the literature. In this systematic review and meta-analysis, conducted in the framework of the international and multicentric FertiTOX project (www.fertitox.com), we therefore assessed the occurrence of suspected subfertility and infertility in patients with leukemia, stratified by sex, leukemia type and treatment modality.

## 2. Methods

### 2.1. Study Design

In May 2024 we conducted a systematic literature review and meta-analysis (see Supplementary Table S1). The study protocol was prospectively registered with PROSPERO (#CRD42024511946) in accordance with the Preferred Reporting Items for Systematic Reviews and Meta-Analyses (PRISMA) guidelines (Supplementary Table S2) [10]. We conducted a comprehensive literature search to identify relevant studies examining the impact of leukemia therapy on fertility outcomes according to the PECO model [11]:

Patients: Girls/women and boys/men with ALL, AML and chronic myeloid leukemia (CML);

Exposure: Radiotherapy, chemotherapy, immunotherapy;

Comparisons: Not applicable;

Outcome: Primary outcome: Infertility, defined as the proportion of patients unable to conceive naturally after cancer treatment; secondary outcome: subfertility, assessed as reduced fertility potential without complete azoospermia/amenorrhea.

Table 1 and Table 2 list the parameters used to define suspected subfertility and suspected infertility in females and males, respectively. A patient was classified as suspected subfertile or suspected infertile if at least one of these parameters fell within the ranges specified below. Although a single parameter, such as anti-Müllerian hormone (AMH), does not fully capture fertility status, we adopted this approach to provide a systematic and comprehensive synthesis of the available literature data. We therefore use the term “suspected” infertility to appropriately reflect this uncertainty.

### 2.2. Information Sources

To identify relevant studies, a comprehensive literature search was conducted in MEDLINE, Embase, Cochrane Database of Systematic Reviews, and Cochrane Central Register of Controlled Trials (CENTRAL). The search strategy was built around three core concepts: (1) leukemia, (2) cancer therapies (including chemotherapy, radiotherapy, immunotherapy, and stem cell therapy), and (3) gonadal function and fertility; studies involving leukemia survivors were also included. A medical information specialist (TK) developed an initial search strategy in Embase and validated it against a predefined set of key references to ensure the inclusion of relevant publications (Supplementary Text S1). Following refinement, the search strategy was adapted for each database using database-specific controlled vocabulary terms and free-text keywords. In all databases, the built-in publication year filter was applied to restrict results to studies published from 2000 onwards. The final search was completed on 13 June 2024. Search results were deduplicated using EndNote according to the Bramer method [12] (Supplementary Text S1). An updated literature search was conducted on 29 June 2026, to identify additional studies published since the initial search. The search identified 29 records (see the search strategy in the Supplementary Materials), of which two met the inclusion criteria [13,14].

One study reported hypergonadotropic hypogonadism in 3.9% of survivors of ALL after mixed treatment modalities [13], while the other reported POI in 88.2% of patients with AML and 93.9% of patients with ALL following HSCT [14]. Both studies were rated as having poor methodological quality according to the Newcastle–Ottawa Scale.

As both studies were consistent with the existing evidence and were of poor methodological quality, their inclusion was not expected to meaningfully affect the pooled estimates or the conclusions of the review. Therefore, an updated meta-analysis was not considered warranted.

### 2.3. Study Selection

In a stepwise approach articles were screened for title, abstract and full text independently by two researchers respectively (NE, JA, MB) using Covidence [15]. Conflicts were resolved by consensus or with the third researcher who was not involved in screening the respective literature.

Articles were evaluated according to the following inclusion and exclusion criteria:

Inclusion criteria: We included human-subject studies published in peer-reviewed journals since 2000, available in English or German and reporting on randomized controlled trials (RCTs), observational (cross-sectional, clinically controlled and cohort studies, both prospective and retrospective), and pre-post studies as well as editorials reporting original data that evaluated the effects of oncologic treatments on gonadal function in leukemia patients (ALL, AML, or CML). Despite its rarity in children, we included CML data to support patient counseling. Eligible participants were females under 40 years of age at fertility evaluation and males of any age, irrespective of treatment phase or disease status, who received chemotherapy, radiotherapy, immunotherapy (including targeted and immune-based agents) or stem cell transplantation. Information on fertility parameters as defined in Table 1 and Table 2 had to be reported at least one year post-treatment completion—CML patients were included even if they had shorter intervals between diagnosis and outcome due to the nature of the disease.

Exclusion criteria: Studies were excluded in case of (systematic) reviews or other meta-analyses, case reports, conference abstracts, educational papers and non-peer-reviewed materials. For analysis of the rate of suspected subfertility or infertility, studies which included fewer than 5 patients of interest or were conducted exclusively in a fertile patient population (e.g. sperm outcome of patients who fathered children) were also excluded. Due to the scarcity of data, for the subgroup analysis of females who underwent ovarian tissue cryopreservation (OTC), smaller studies were included, except for single case studies. Studies were also excluded if more than 10% of the total cohort failed to meet the inclusion criteria.

### 2.4. Data Extraction

Relevant data were extracted independently by two out of three researchers (NE, JA, MB) considering study characteristics (year of publication, study type, study duration), patient characteristics (type of leukemia, sex, age at diagnosis and outcome), treatment details (type of treatment, including dosage and location), as well as fertility outcomes (Table 1 and Table 2, timing of outcome measure). Conflicts were resolved by consensus or the third reviewer who was not involved in the extraction. Any differences in units were harmonized through conversion.

### 2.5. Data Synthesis and Statistical Analyses

Analyses were conducted by an independent researcher (JP). Quantitative data were pooled using random-effects meta-analysis models to account for between-study heterogeneity. Prevalence of infertility and subfertility was calculated by dividing the number of patients meeting the predefined diagnostic criteria (Table 1 and Table 2) by the total number of at-risk patients reported in each individual study. Heterogeneity was assessed using the I^2^ statistic, with I^2^ > 50% indicating substantial heterogeneity. Prespecified subgroup analyses were performed by leukemia subtype (ALL, AML, CML), treatment modality, and patient sex to explore potential effect modifiers. All statistical analyses were conducted using the “metafor” package (version 4.8-0) within R statistical software (version R 4.5.2) using R studio (version 2025.05.1+513).

### 2.6. Quality Assessment

The methodological quality and risk of bias of the 42 studies were independently assessed by two researchers (NE, JA, MB) using the Newcastle–Ottawa Scale [16]. Discrepancies were resolved by consensus or through consultation with the third researcher. The scoring of individual studies was based on three parameters: subject selection (0–4 stars), comparability (0–2 stars), and study outcome (0–3 stars). The scoring was as follows: good quality (=3 or 4 stars in selection AND 1 or 2 stars in comparability AND 2 or 3 stars in outcome/exposure), fair quality (=2 stars in selection AND 1 or 2 stars in comparability AND 2 or 3 stars in outcome/exposure), and poor quality (=0 or 1 star in selection OR 0 stars in comparability OR 0 or 1 stars in outcome/exposure).

## 3. Results

### 3.1. Studies Identified and Characteristics of Included Studies

**Figure 1:**
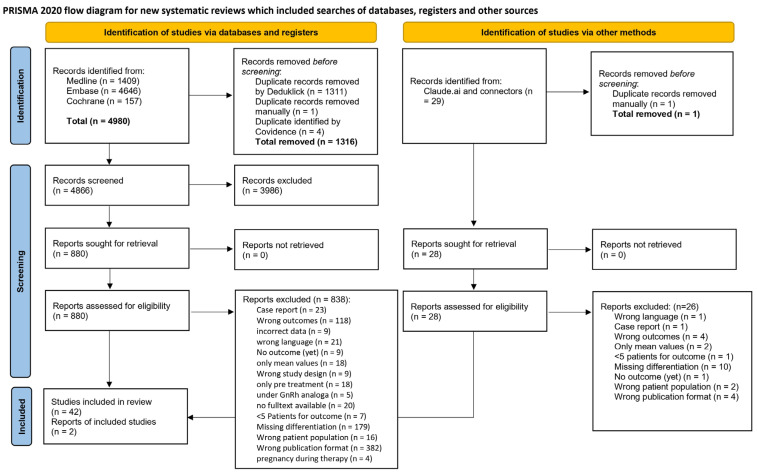
PRISMA Chart on inclusion of studies.

Our search strategy prompted 6182 studies. Following the screening process 42 studies were identified for analysis, including seven studies addressing post-cancer treatment fertility following OTC (Figure 1) [8,17,18,19,20,21,22,23,24,25,26,27,28,29,30,31,32,33,34,35,36,37,38,39,40,41,42,43,44,45,46,47,48,49,50,51,52,53,54,55,56,57].

The study designs included retrospective studies (*n* = 7), retrospective longitudinal studies (*n* = 24), surveys (*n* = 4), cross-sectional studies (*n* = 2), and prospective studies (*n* = 5). One publication contributed data from both a retrospective longitudinal analysis (a) and a survey component (b) [23]. Overall two studies were rated with good methodological quality, one with fair and 39 with poor quality. Poor ratings were primarily related to the absence of control groups and limited comparability of fertility outcomes when control groups were available (see Supplementary Table S3 for details on the quality assessment of included studies).

Among the 42 included studies, sample sizes ranged from 6 to 551 patients for the main analysis; for the subgroup analysis for OTC sample sizes ranged from 4 to 18 patients. Overall, data from 2048 patients with leukemia were analyzed, including 1376 females and 672 males. The predominance of female participants in the overall sex distribution is largely attributable to one survey study that included 551 female participants [23]. Reported diagnoses included ALL, AML, CML, and acute biphenotypic leukemia. In many studies, the exact distribution of leukemia subtypes was not specified and/or was described only as “acute leukemia” or “mixed leukemia cohort.” The treatment period spanned from 1964 to the present. The median age at diagnosis was 12.75 years (range: 1 month to 39 years among females and up to 45 years among males). At follow-up, ages ranged from 3 to 39 years in females and up to 57 years in males. The duration of follow-up ranged from 3 to 41 years. Treatment characteristics were heterogeneous, and detailed therapeutic data were frequently unavailable. The majority of patients received multi-agent chemotherapy, with some additionally undergoing irradiation and/or hematopoietic stem cell transplantation. The methods and timing of fertility assessment varied substantially across the included studies (see Supplementary Table S4 for details of included studies).

The studies were conducted across diverse geographic regions (see Supplementary Table S4).

The meta-analysis revealed considerable between-study heterogeneity (up to I^2^ = 91%). In the following, results are presented stratified by sex (females and males) across the included studies.

### 3.2. Prevalence of Infertility and Subfertility in Females

**Figure 2:**
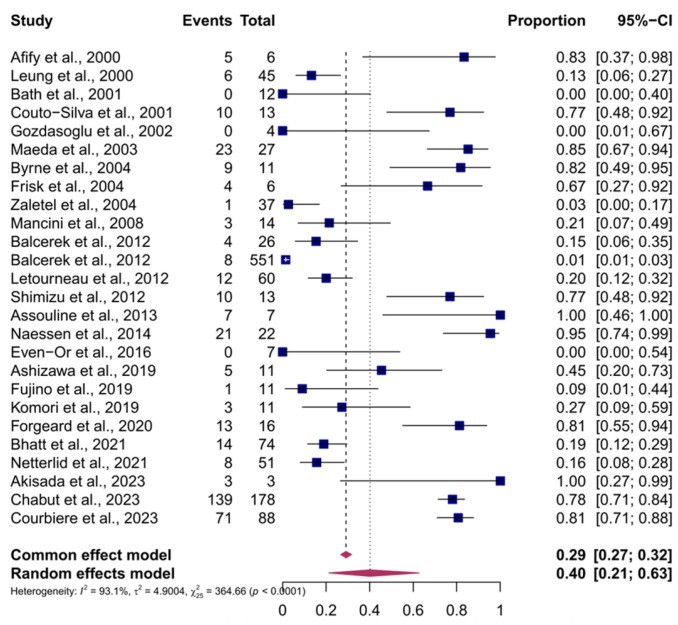
Prevalence of infertility in females with leukemia after all types of treatments. Blue box: interquartile range (IQR). Horizontal line: median. Diamond: mean. References in order top to bottom [18,19,20,21,23,24,25,27,28,29,30,31,32,34,35,36,40,41,42,43,44,46,48,54,57].

Overall, using a random-effects model, the pooled prevalence of infertility was 40% (95% CI, 21–63%) among females with a history of leukemia (Figure 2), excluding studies with prior OTC.

This rate differed between leukemia diagnoses and affected 66% (40–85%) of females following a diagnosis of ALL, 57% (29–81%) following AML, and 96% (21–100%) after CML (Supplementary Figures S1–S3). The different treatment modalities used in leukemia treatment had a broad range of gonadal effects, and were lowest in those receiving standard treatment with polychemotherapy (4% (95% CI, 0–65%)).

**Figure 3:**
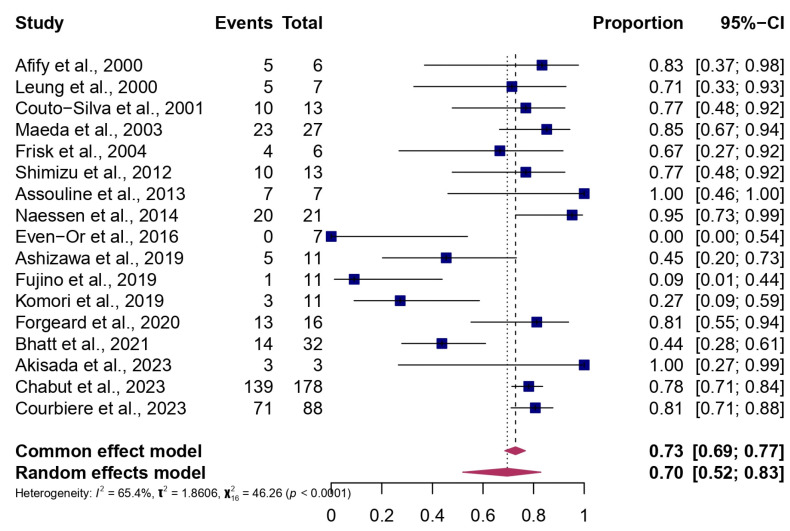
Prevalence of infertility in females with leukemia after HSCT. Blue box: interquartile range (IQR). Horizontal line: median. Diamond: mean. References in order top to bottom [18,19,20,21,25,28,29,30,31,32,34,35,40,42,43,46,54].

The prevalence of infertility was highest after HSCT (70% (95% CI, 52–83%, see Figure 3); without TBI = 70% (95% CI, 29–93%), with TBI = 67% (95% CI, 52–79%)).

Prevalence of subfertility was reported for 12% (95% CI, 1–60%) of females across all leukemia groups (Supplementary Figure S4).

In the subgroup of females post-OTC (*n* = 65, mean age at diagnosis 16.13 (2–39) years) infertility occurred in 91% (95% CI, 67–98%), at a follow-up time ranging from 20.4 months to 10.5 years [8,17,38,39,51,53,55]. In 6 of the 7 studies [8,17,39,51,53,55], OTC was performed before HSCT, one of which included patients with previous alkylating agent exposure prior to salvage HSCT [17]. In one study therapy details were not reported [38]. OTC in prepubertal girls was usually performed through unilateral oophorectomy, in some cases through partial resection of bilateral ovarian tissue (Supplementary Figure S5).

### 3.3. Prevalence of Infertility and Subfertility in Males

**Figure 4:**
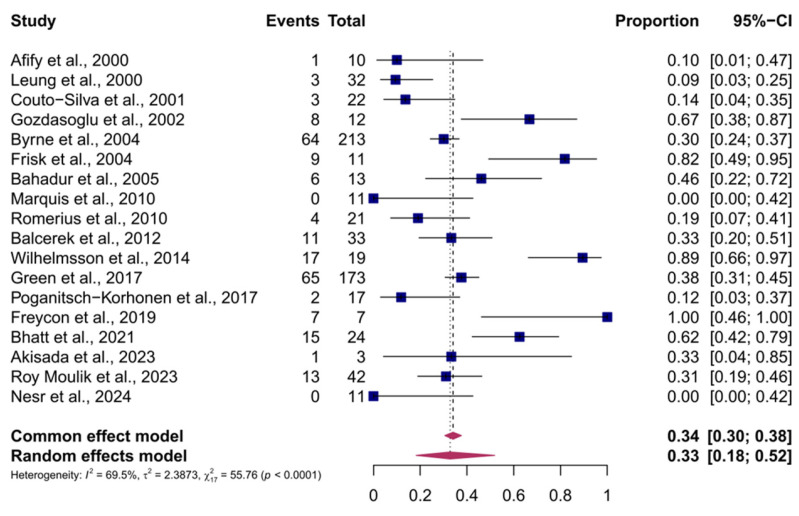
Prevalence of infertility in males with leukemia after all types of treatments. Blue box: interquartile range (IQR). Horizontal line: median. Diamond: mean. References in order top to bottom [18,19,22,23,25,26,30,33,34,36,37,42,45,47,49,50,52,56].

In male leukemia patients, infertility occurred in 33% (95% CI, 18–52%; Figure 4).

**Figure 5:**
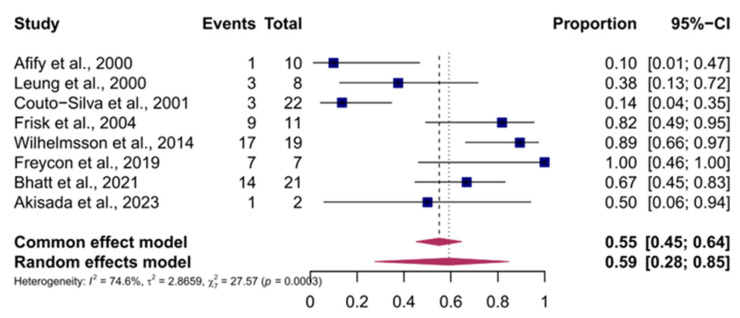
Prevalence of infertility in males with leukemia after HSCT. Blue box: interquartile range (IQR). Horizontal line: median. Diamond: mean. References in order top to bottom [18,19,25,30,33,34,42,56].

This was highest in ALL (27% (95% CI, 12–50%), with lower occurrences for AML (14% (95% CI, 3–51%)) and CML (11% (95% CI, 1–74%)); Supplementary Figures S6–S8). Infertility occurred in 2% (95% CI, 0–81%) of males treated with polychemotherapy. Following HSCT, the prevalence reached 59% (95% CI, 28–85%, see Figure 5), without TBI = 57% (95% CI, 26–84%); with TBI = 70% (95% CI, 21–95%).

Testicular irradiation conferred the greatest risk of infertility (92% (95% CI, 59–99%; Supplementary Figure S9).

Subfertility was seen in 20% (95% CI, 13–29%) of males across all leukemia diagnoses (Supplementary Figure S10).

## 4. Discussion

### 4.1. Main Results

This systematic review and meta-analysis provides the first comprehensive, quantitative synthesis of fertility outcomes in leukemia survivors, stratified by treatment modality, sex, and leukemia subtype. Overall, more than one third (37%, 95% CI 23–54%) of leukemia survivors experienced clinical infertility, substantially exceeding estimates for the general population (12.6–17.5%) [58]. Infertility risk varied markedly with treatment intensity and was further modulated by sex, age at exposure, and leukemia subtype. Given that leukemia treatment usually requires immediate initiation, FP is frequently constrained by young age, time pressure, and an unpredictable disease course. Reliable estimates of treatment-related fertility risk are therefore of high clinical relevance [58].

Infertility prevalence varied markedly especially by treatment intensity, with additional modulation by sex, age at exposure and leukemia subtype. Patients treated with polychemotherapy alone generally showed low infertility and subfertility rates, supporting the incorporation of structured reproductive monitoring into routine long-term follow-up. However, subtle ovulatory disorders may still occur [24]. In contrast, HSCT emerged as the dominant risk factor. Across studies, infertility rates approached 68%, with consistently higher vulnerability in females (~65%) than in males (~40%), particularly following myeloablative conditioning (MAC) [6,59]. Conditioning regimens used in leukemia patients commonly include busulfan, cyclophosphamide, fludarabin, and melphalan, with or without TBI, of which busulfan appears to have particularly strong gonadotoxic effects [43,60]. Although reduced-intensity chemotherapy regimens show higher rates of pubertal recovery as demonstrated in a marked increase in spontaneous puberty compared with MAC (90% vs. 56% of girls) [59], the risk of premature ovarian insufficiency (POI) remains high (pooled prevalence of up to 86%) [21,31,54]. Due to limited reporting, differentiation by individual conditioning regimens was not feasible in this meta-analysis.

Recovery from gonadal failure, the most common endocrine complication after HSCT [28], is rare and strongly age-dependent, with younger patients showing higher recovery potential [6,28]. Additional risk factors include disease relapse, exposure to alkylating agents and cranial, pelvic, or TBI [6,61], as well as prior OTC as a significant risk factor for the development of POI [28]. Nearly 50% of girls who had not yet reached puberty at HSCT required HRT to initiate pubertal development [62].

Irradiation demonstrated a dose-dependent effect on fertility, with the highest infertility rates observed after testicular irradiation (92%) [33] and TBI (66%) [20,29]. These findings are consistent with established gonadotoxic thresholds. Testicular doses >6 Gy result in permanent azoospermia in >90% of patients, while POI typically occurs after >10–15 Gy depending on age at exposure, explaining observed sex-specific differences. Gonadal shielding is an established strategy of risk mitigation but raises concerns regarding leukemic sanctuary sites. Only one study reported on ovarian shielding within a MAC regimen which included 10–12 Gy TBI, reducing ovarian exposure to 2–3 Gy and showing reduced ovarian toxicity without increased relapse in standard-risk patients [20]. Reduced-toxicity MAC with 8 Gy TBI may lower POI risk, particularly in prepubertal patients, though long-term reproductive outcomes remain unclear [40]. Beyond gonadal damage, irradiation may also compromise uterine structures and vascularization, impairing implantation and pregnancy outcomes. Childhood TBI causes irreversible uterine volume reduction (43% after alkylating agent–based regimens and 75% after TBI), despite adequate HRT, resulting in low live birth rates (11%) and high miscarriage rates [29]. In males, TBI is associated with reduced testicular volume [30]. Data on CRT were limited; nevertheless, evidence indicates a substantial reduction in female fertility at any dose of CRT, reaching only about one-quarter of that of their sibling controls especially when exposure occurs within two years of menarche [27]. In males, infertility risk mainly increased after high-dose CRT (>24 Gy) before age nine [26], whereas lower-dose CRT (<20 Gy or 20–26 Gy) seems not to have an effect [37]. Children diagnosed at 5–9 years and those treated with 8–12 or ≥12 g/m^2^ cyclophosphamide-equivalent dose (CED) had a higher risk of impaired spermatogenesis than younger patients or those receiving >0–<4 g/m^2^ CED [37].

Our meta-analysis revealed clear sex-specific vulnerabilities. Males showed higher susceptibility to infertility after chemotherapy-only regimens (18% vs. 6% in females), whereas females were more affected after HSCT (70% vs. 59% in males). Post-HSCT pregnancy rates remained low across studies (5–25%) [21]. These findings are clinically relevant. In females, chemotherapy or radiation-induced depletion of the finite oocyte pool may cause irreversible POI and drastically shorten the reproductive window [7]. FP options are frequently limited by prepubertal age and urgency to initiate treatment, precluding oocyte cryopreservation in many cases [63]. Gonadotropin-releasing hormone agonists may reduce menstrual bleeding and possibly ovarian damage, but are insufficient as a standalone strategy [63,64]. In children, their use is not approved and their effectiveness in adolescents is limited [63,64]. Prior to HSCT or in high-risk settings, OTC may be considered but remains experimental and highly controversial due to potential leukemic contamination [65]. Although reported recurrence risk appears low, it cannot be fully excluded [64,66], even in patients with minimal residual disease (MRD) negativity in blood and bone marrow [65]. Therefore, OTC requires careful shared decision-making, particularly in standard-risk patients. Moreover, OTC itself may reduce ovarian reserve and tissue retrieved after chemotherapy may exhibit compromised follicular quality [17]. The latter was measured by Abir et al. (AMH 24 h before and 24 h after unilateral oophorectomy), showing a distinctive impact of OTC itself on fertility through a decrease in AMH levels [17]. Live birth rates after OTC are ~27% and are strongly age-dependent (<35 years at ovarian tissue retrieval: 28%; >35 years: 17%) [67]. Experimental approaches to bypass the risk of recurrence via ovarian tissue transplantation, such as in vitro follicle maturation or artificial ovaries containing primordial follicles derived from cryopreserved tissue, remain in preclinical development [68,69]. When primary FP is not feasible, secondary strategies after treatment (“second-stop concept”) should be pursued [64]. This includes assessment of ovarian reserve ≥1 year post-treatment using AMH levels and, whenever available, antral follicle count. Patients with adequate reserve require monitoring only, while those with reduced reserve but regular cycles should be offered oocyte cryopreservation where possible. HRT is indicated for incipient POI [64,70]. Although FP success rates after cancer treatment are lower than in healthy peers, it often represents the only available option [64,70].

In males, the highest infertility risk is associated with testicular irradiation, high-dose alkylating agents, and CRT (24 Gy) before age 10 [22,26,45,50]. Testicular irradiation had the most detrimental effect (92% infertility), often causing hypergonadotropic hypogonadism requiring androgen replacement therapy [45,50]. Gonadotoxic chemotherapy leads to variable degrees of azoospermia and oligozoospermia [22], with Inhibin B, FSH and testicular volume serving as predictive markers [56]. Fertility impairment may already be present at diagnosis, likely reflecting disease-related gonadal dysfunction. Recovery of spermatogenesis is possible and depends on disease and baseline sperm quality [22,50]. Recovery is common in patients not undergoing HSCT [56] with over 70% treated with low-dose alkylating agents (CED ≤ 7500 mg/m^2^) recovering spermatogenesis, compared to 10–50% after higher doses [49,56,71] and typically over 5–8 years [49,56,71]. Yet, we report increased infertility rates also in the polychemotherapy-only group compared to that reported for the general population. Semen cryopreservation is therefore strongly recommended whenever feasible [64]. In azoospermic patients, testicular sperm extraction (TESE) may retrieve viable sperm in ~50% of cases [72]. In prepubertal boys, testicular tissue cryopreservation remains experimental [72]. Follow-up—especially after high-risk treatment—should not rely solely on hormonal assessment but include repeat semen analyses, which remain the gold standard of fertility evaluation in males [64,72]. Tyrosine kinase inhibitors have transformed CML treatment, reducing the need for HSCT [52]. While short-term data suggest minimal effect on sperm parameters [47], long-term follow-up shows reduced sperm quality in >40% of adolescents (azoospermia in 31%; delayed puberty in 15%), particularly in those diagnosed before age 5 [52].

Irrespective of sex, given the unpredictability of treatment escalation and fertility risk even with standard therapy, early and repeated oncofertility counseling is essential for informed family planning of patients affected—ideally at diagnosis, at treatment completion, and during long-term follow-up. Insufficient counseling negatively affects long-term psychological well-being [44], while FP itself may improve emotional coping regardless of later use [44]. Oncofertility counseling should also address alternative paths to parenthood within national legal frameworks such as oocyte, embryo or sperm donation, surrogacy, and include adoption and foster care.

Patients should additionally be informed about the risk and symptoms of early menopause or androgen deficiency beyond infertility alone [41]. These conditions are associated with osteopenia/osteoporosis, sarcopenia, metabolic and cardiovascular disease, climacteric symptoms, sexual dysfunction and reduced quality of life, among others [41,73,74]. Providing patients with adequate guidance on lifestyle modifications and HRT is a key component to alleviate symptoms and enhance quality of life [41,73,74].

### 4.2. Methodological Considerations and Limitations

Fertility assessment methods varied considerably across studies. Only studies with predefined fertility criteria and sufficient information were included. In females, AMH was most frequently reported, whereas combined assessment using AMH and FSH was rarely available. Furthermore, AMH may already be reduced at diagnosis and up to one year after cancer treatment, with little potential for recovery [75]. Similarly, menstrual irregularities, including amenorrhea, may persist up to 1-year post-treatment [73,75] and warrant evaluation if newly occurring. Although a single parameter, such as AMH, does not fully reflect fertility status, the available data did not allow for a more comprehensive definition of infertility based on multiple parameters. We therefore use the term “suspected” infertility to reflect this limitation. Comprehensive fertility assessment has only recently been introduced into routine clinical care, and older studies often lacked relevant reproductive endpoints.

However, it should be noted that a single AMH value does not sufficiently reflect a woman’s reproductive capacity and should be considered longitudinally. A detailed fertility comprehensive assessment—including history, risk factors, cycle parameters, gynecological examination, ultrasound including antral follicle count, ovarian and uterine morphology in females as well as semen analyses in males, and hormone profiling—provides more accurate risk prediction [63,64,73]. Survivorship guidelines recommend risk-adapted (annually to 5-yearly) endocrine follow-up in both sexes [73]. In males, heterogenous outcome measures ranging from hormonal parameters to semen analyses contributed to inter-study variability.

The heterogeneity of available data highlights the need for standardized, longitudinal data, such as the ongoing prospective FertiTOX study, to support individualized counseling and family planning. Novel therapies such as immunotherapies and CAR-T cells were not included in the present analysis. While studies indicate some degree of hypospermatogenesis and aspermatogenesis in men, and damage to ovarian reserves, recurrent pregnancy loss, and implantation failure in women, this area remains underexplored [9]. Entering a new era of treatment options for leukemia patients, prospective randomized studies are needed to address the gonadotoxic potential of these promising new therapies.

Our meta-analysis has several limitations. First, included studies used heterogeneous definitions of infertility (biochemical vs. clinical), limiting comparability (I^2^ = 91%). Second, the timing of fertility assessment as well as follow-up duration varied substantially and longer-term outcomes beyond 20 years remain underrepresented. Third, the preponderance of retrospective studies may introduce selection bias, particularly regarding fertility intentions and outcomes. Fourth, modern treatment protocols (CAR-T cell therapy, targeted agents) are not represented in the included studies, limiting applicability to contemporary leukemia management. The generalizability of our findings is limited by the heterogeneity of the available data, partly incomplete reporting of treatment details, and variability in follow-up assessments and timing. An additional limiting factor is variability in treatment protocols over time, as some included studies reflect anticancer treatment regimens administered several decades ago.

## 5. Conclusions

In conclusion, our findings underscore the need for early and repeated oncofertility counseling in leukemia patients, especially in light of the unpredictability of treatment escalation and the risk of gonadal impairment even with standard therapy. Prospective cohort studies with standardized gonadal function assessments pre-, during and post-treatment, long-term fertility outcome ascertainment, and incorporation of novel treatments are urgently needed. In addition, the psychological and quality-of-life consequences of infertility in leukemia survivors require dedicated investigation, and implementation science studies examining barriers to fertility preservation counseling and uptake, particularly in resource-limited settings, are critical. Counseling regarding future reproductive potential and fertility preservation options should begin at diagnosis and continue at the end of treatment and throughout long-term follow-up care.

## Figures and Tables

**Table 1:** Outcome parameters used to define suspected subfertility and suspected infertility in females.

Parameter	Suspected Subfertility	Suspected Infertility
AMH	<0.5 ng/mL	not detectable
FSH	10–25 IU/L	>25 IU/L
menstrual cycle	oligomenorrhoea note: hormonal contraception	amenorrhea note: hormonal contraception
POI		yes
hormone replacement therapy		yes
WHO definition: did not conceive after >12 months of trying		yes
hypergonadotropic hypogonadism		yes
AFC	<5 AFC note: hormonal contraception	

AFC: antral follicle count. AMH: anti-mullerian hormone. FSH: follicle-stimulating hormone. POI: premature ovarian insufficiency. WHO: World Health Organization.

**Table 2:** Outcome parameters to define suspected subfertility and suspected infertility in males.

Parameter	Suspected Subfertility	Suspected Infertility
FSH	10–25 IU/L	>25 IU/L
semen analyses	oligozoospermia/asthenozoospermia	azoospermia
Inhibin B	decreased if lower than normal range (125–215 pg/mL)	<80 pg/mL and FSH of >10 IU/L
Hormone replacement therapy		yes
WHO definition: did not conceive after >12 months of trying		yes
hypergonadotropic hypogonadism		yes

FSH: follicle-stimulating hormone, WHO: World Health Organization.

## Data Availability

The datasets generated during and/or analyzed during the current study are available from the corresponding author on reasonable request.

## Supplementary Materials

The following supporting information can be downloaded at: https://www.mdpi.com/article/10.3390/cancers18152455/s1, Text S1: Search strategy. Figure S1: Prevalence of infertility in females with ALL; Figure S2: Prevalence of infertility in females with AML; Figure S3: Prevalence of infertility in females with CML; Figure S4: Prevalence of subfertility in females following all types of treatments; Figure S5: Post-treatment infertility in female leukemia patients after ovarian tissue cryopreservation; Figure S6: Prevalence of infertility in males with ALL; Figure S7: Prevalence of infertility in males with AML; Figure S8: Prevalence of infertility in males with CML; Figure S9: Prevalence of infertility in males after testicular irradiation; Figure S10: Prevalence of subfertility in males following all types of treatments; Table S1: Search Strategy; Table S2: PRISMA 2020 checklist; Table S3: Quality assessment of included studies; Table S4: Characteristics of included studies.

## References

[1] Deutsches Kinderkrebsregister. Jahresbericht. https://www.kinderkrebsregister.de/dkkr/ergebnisse/jahresberichte/jahresbericht-2022.html.

[2] Robert Koch-Institut, Abteilung für Epidemiologie und Gesundheitsmonitoring Krebsdaten Deutschland. https://www.krebsdaten.de/Krebs/DE/Home/homepage_node.html.

[3] Stelljes M., Marks D.I., Giebel S., Sureda A., Corbacioglu S., Greco R., Kröger N., Carreras E. (2024). Acute Lymphoblastic Leukemia in Adults. The EBMT Handbook: Hematopoietic Cell Transplantation and Cellular Therapies.

[4] Peters C., Locatelli F., Bader P., Sureda A., Corbacioglu S., Greco R., Kröger N., Carreras E. (2024). Acute Lymphoblastic Leukaemia in Children and Adolescents. The EBMT Handbook: Hematopoietic Cell Transplantation and Cellular Therapies.

[5] van Dijk M., Berg M.H.V.D., Overbeek A., Lambalk C.B., Heuvel-Eibrink M.M.V.D., Tissing W.J., Kremer L.C., van der Pal H.J., Loonen J.J., Versluys B. (2018). Reproductive intentions and use of reproductive health care among female survivors of childhood cancer. Hum. Reprod..

[6] Vidal A., Bora C., Jarisch A., Pape J., Weidlinger S., Karrer T., von Wolff M. (2025). Impact of haematopoietic stem cell transplantation for benign and malignant haematologic and non-haematologic disorders on fertility: A systematic review and meta-analysis. Bone Marrow Transplant..

[7] Pfitzer C., Orawa H., Balcerek M., Langer T., Dirksen U., Keslova P., Zubarovskaya N., Schuster F.R., Jarisch A., Strauss G. (2014). Dynamics of fertility impairment and recovery after allogeneic haematopoietic stem cell transplantation in childhood and adolescence: Results from a longitudinal study. J. Cancer Res. Clin. Oncol..

[8] Biasin E., Salvagno F., Berger M., Nesi F., Quarello P., Vassallo E., Evangelista F., Marchino G.L., Revelli A., Benedetto C. (2015). Ovarian tissue cryopreservation in girls undergoing haematopoietic stem cell transplant: Experience of a single centre. Bone Marrow Transplant..

[9] Caserta S., Cancemi G., Murdaca G., Stagno F., Di Gioacchino M., Gangemi S., Allegra A. (2024). The Effects of Cancer Immunotherapy on Fertility: Focus on Hematological Malignancies. Biomedicines.

[10] Page M.J., McKenzie J.E., Bossuyt P.M., Boutron I., Hoffmann T.C., Mulrow C.D., Shamseer L., Tetzlaff J.M., Akl E.A., Brennan S.E. (2021). The PRISMA 2020 statement: An updated guideline for reporting systematic reviews. BMJ.

[11] Morgan R.L., Whaley P., Thayer K.A., Schünemann H.J. (2018). Identifying the PECO: A framework for formulating good questions to explore the association of environmental and other exposures with health outcomes. Environ. Int..

[12] Bramer W.M., Giustini D., de Jonge G.B., Holland L., Bekhuis T. (2016). De-duplication of database search results for systematic reviews in EndNote. J. Med. Libr. Assoc..

[13] Karakaş H., Tarçın G., Bayramoğlu E., Turan H., Ocak S., Turan V., Evliyaoğlu O., Tiraje T., Apak H., Ercan O. (2026). Long-Term endocrine outcomes with special emphasis on the gonadal impact of acute lymphoblastic leukemia treatment in females. Ann. Hematol..

[14] Hou Y., Li Z., Bai L., Li S., Ai J., Cheng C., Tian L., Cheng Y., Wang J. (2025). Fertility assessment in long-term young female survivors with hematological disease after allogeneic hematopoietic cell transplantation: A single-center real-life cross-sectional study. Ann. Hematol..

[15] Covidence Systematic Review Software. Melbourne, Australia: Veritas Health Innovation. https://www.covidence.org.

[16] Wells G., Shea B., O’Connell D., Peterson j Welch V., Losos M., Tugwell P. (2000). The Newcastle–Ottawa Scale (NOS) for Assessing the Quality of Non-Randomized Studies in Meta-Analysis. https://ohri.ca/en/who-we-are/core-facilities-and-platforms/ottawa-methods-centre/newcastle-ottawa-scale.

[17] Abir R., Ben-Aharon I., Garor R., Yaniv I., Ash S., Stemmer S., Ben-Haroush A., Freud E., Kravarusic D., Sapir O. (2016). Cryopreservation of in vitromatured oocytes in addition to ovarian tissue freezing for fertility preservation in paediatric female cancer patients before and after cancer therapy. Hum. Reprod..

[18] Afify Z., Shaw P.J., Clavano-Harding A., Cowell C.T. (2000). Growth and endocrine function in children with acute myeloid leukaemia after bone marrow transplantation using busulfan/cyclophosphamide. Bone Marrow Transplant..

[19] Akisada H., Hasegawa M., Ishihara T., Akisada N., Ochi S., Nogami K. (2023). Endocrine late effects in survivors of infantile acute lymphoblastic leukemia. Clin. Pediatr. Endocrinol..

[20] Ashizawa M., Akahoshi Y., Nakano H., Kawamura S., Takeshita J., Yoshino N., Misaki Y., Yoshimura K., Gomyo A., Tamaki M. (2019). Updated Clinical Outcomes of Hematopoietic Stem Cell Transplantation Using Myeloablative Total Body Irradiation with Ovarian Shielding to Preserve Fertility. Biol. Blood Marrow Transplant..

[21] Assouline E., Crocchiolo R., Prebet T., Broussais F., Coso D., Gamerre M., Vey N., Blaise D., Courbiere B. (2013). Impact of reduced-intensity conditioning allogeneic stem cell transplantation on women’s fertility. Clin. Lymphoma Myeloma Leuk..

[22] Bahadur G., Ozturk O., Muneer A., Wafa R., Ashraf A., Jaman N., Patel S., Oyede A.W., Ralph D.J. (2005). Semen quality before and after gonadotoxic treatment. Hum. Reprod..

[23] Balcerek M., Reinmuth S., Hohmann C., Keil T., Borgmann-Staudt A. (2012). Suspected infertility after treatment for leukemia and solid tumors in childhood and adolescence. Dtsch. Arztebl. Int..

[24] Bath L.E., Anderson R.A., Critchley H.O., Kelnar C.J., Wallace W.H. (2001). Hypothalamic-pituitary-ovarian dysfunction after prepubertal chemotherapy and cranial irradiation for acute leukaemia. Hum. Reprod..

[25] Bhatt N.S., Baassiri M.J., Liu W., Bhakta N., Chemaitilly W., Ehrhardt M.J., Inaba H., Krull K., Ness K.K., Rubnitz J.E. (2021). Late outcomes in survivors of childhood acute myeloid leukemia: A report from the St. Jude Lifetime Cohort Study. Leukemia.

[26] Byrne J., Fears T.R., Mills J.L., Zeltzer L.K., Sklar C., Meadows A.T., Reaman G.H., Robison L.L. (2004). Fertility of long-term male survivors of acute lymphoblastic leukemia diagnosed during childhood. Pediatr. Blood Cancer.

[27] Byrne J., Fears T.R., Mills J.L., Zeltzer L.K., Sklar C., Nicholson H.S., Haupt R., Reaman G.H., Meadows A.T., Robison L.L. (2004). Fertility in women treated with cranial radiotherapy for childhood acute lymphoblastic leukemia. Pediatr. Blood Cancer.

[28] Chabut M., Schneider P., Courbiere B., Saultier P., Bertrand Y., Tabone M.D., Pochon C., Ducassou S., Paillard C., Gandemer V. (2023). Ovarian Function and Spontaneous Pregnancy After Hematopoietic Stem Cell Transplantation for Leukemia Before Puberty: An, L.E.A. Cohort Study. Transplant. Cell. Ther..

[29] Courbiere B., Drikes B., Grob A., Hamidou Z., Saultier P., Bertrand Y., Gandemer V., Plantaz D., Plat G., Poirée M. (2023). The uterine volume is dramatically decreased after hematopoietic stem cell transplantation during childhood regardless of the conditioning regimen. Fertil. Steril..

[30] Couto-Silva A.C., Trivin C., Thibaud E., Esperou H., Michon J., Brauner R. (2001). Factors affecting gonadal function after bone marrow transplantation during childhood. Bone Marrow Transplant..

[31] Even-Or E., Ben-Haroush A., Yahel A., Yaniv I., Stein J. (2016). Fertility After Treatment with High Dose Melphalan in Women with Acute Myelogenous Leukemia. Pediatr. Blood Cancer.

[32] Forgeard N., Jestin M., Vexiau D., Chevillon F., Ricadat E., Peffault de Latour R., Robin M., Sicre de Fontbrune F., Xhaard A., Michonneau D. (2021). Sexuality- and Fertility-Related Issues in Women after Allogeneic Hematopoietic Stem Cell Transplantation. Transplant. Cell. Ther..

[33] Freycon F., Casagranda L., Trombert-Paviot B. (2019). The impact of severe late-effects after 12 Gy fractionated total body irradiation and allogeneic stem cell transplantation for childhood leukemia (1988–2010). Pediatr. Hematol. Oncol..

[34] Frisk P., Arvidson J., Gustafsson J., Lönnerholm G. (2004). Pubertal development and final height after autologous bone marrow transplantation for acute lymphoblastic leukemia. Bone Marrow Transplant..

[35] Fujino H., Ishida H., Iguchi A., Onuma M., Kato K., Shimizu M., Yasui M., Fujisaki H., Hamamoto K., Washio K. (2019). High rates of ovarian function preservation after hematopoietic cell transplantation with melphalan-based reduced intensity conditioning for pediatric acute leukemia: An analysis from the Japan Association of Childhood Leukemia Study (JACLS). Int. J. Hematol..

[36] Gözdaşoğlu S., Aksoylar S., Berberoğlu M., Öcal G., Adıyaman P., Çavdar A.O., Babacan E., Ünal E., Taçyıldız N., Yavuz G. (2002). Endocrinologic Late Effects of Chemoradiotherapy in Pediatric Acute Leukemia. Turk. J. Haematol..

[37] Green D.M., Zhu L., Wang M., Chemaitilly W., Srivastava D., Kutteh W.H., Ke R.W., Sklar C.A., Pui C.H., Kun L.E. (2017). Effect of cranial irradiation on sperm concentration of adult survivors of childhood acute lymphoblastic leukemia: A report from the St. Jude Lifetime Cohort Study†. Hum. Reprod..

[38] Jadoul P., Guilmain A., Squifflet J., Luyckx M., Votino R., Wyns C., Dolmans M.M. (2017). Efficacy of ovarian tissue cryopreservation for fertility preservation: Lessons learned from 545 cases. Hum. Reprod..

[39] Jensen A.K., Rechnitzer C., Macklon K.T., Ifversen M.R., Birkebæk N., Clausen N., Sørensen K., Fedder J., Ernst E., Andersen C.Y. (2017). Cryopreservation of ovarian tissue for fertility preservation in a large cohort of young girls: Focus on pubertal development. Hum. Reprod..

[40] Komori K., Hirabayashi K., Morita D., Hara Y., Kurata T., Saito S., Tanaka M., Yanagisawa R., Sakashita K., Koike K. (2019). Ovarian function after allogeneic hematopoietic stem cell transplantation in children and young adults given 8-Gy total body irradiation-based reduced-toxicity myeloablative conditioning. Pediatr. Transplant..

[41] Letourneau J.M., Ebbel E.E., Katz P.P., Oktay K.H., McCulloch C.E., Ai W.Z., Chien A.J., Melisko M.E., Cedars M.I., Rosen M.P. (2012). Acute ovarian failure underestimates age-specific reproductive impairment for young women undergoing chemotherapy for cancer. Cancer.

[42] Leung W., Hudson M.M., Strickland D.K., Phipps S., Srivastava D.K., Ribeiro R.C., Rubnitz J.E., Sandlund J.T., Kun L.E., Bowman L.C. (2000). Late effects of treatment in survivors of childhood acute myeloid leukemia. J. Clin. Oncol..

[43] Maeda N., Kato K., Matsuyama T., Kojima S., Ohyama K. (2003). High-dose busulfan is a major risk factor for ovarian dysfunction in girls after stem cell transplantation. Clin. Pediatr. Endocrinol..

[44] Mancini J., Rey D., Préau M., Malavolti L., Moatti J.P. (2008). Infertility induced by cancer treatment: Inappropriate or no information provided to majority of French survivors of cancer. Fertil. Steril..

[45] Marquis A., Kuehni C.E., Strippoli M.P., Kühne T., Brazzola P., Swiss Pediatric Oncology Group (2010). Sperm analysis of patients after successful treatment of childhood acute lymphoblastic leukemia with chemotherapy. Pediatr. Blood Cancer.

[46] Naessén S., Bergström I., Ljungman P., Landgren B.M. (2014). Long-term follow-up of bone density, general and reproductive health in female survivors after treatment for haematological malignancies. Eur. J. Haematol..

[47] Nesr G., Claudiani S., Milojkovic D., Innes A., Fernando F., Caballes I., Mungozi P., Szydlo R., Lovato S., Jayasena C. (2024). Effect of tyrosine kinase inhibitors on male fertility in patients with chronic phase chronic myeloid leukemia. Leuk. Lymphoma.

[48] Netterlid A., Mörse H., Giwercman A., Henic E., Åkesson K.E., Erfurth E.M., Elfving M. (2021). Premature ovarian failure after childhood cancer and risk of metabolic syndrome: A cross-sectional analysis. Eur. J. Endocrinol..

[49] Poganitsch-Korhonen M., Masliukaite I., Nurmio M., Lähteenmäki P., van Wely M., van Pelt A.M.M., Jahnukainen K., Stukenborg J.B. (2017). Decreased spermatogonial quantity in prepubertal boys with leukaemia treated with alkylating agents. Leukemia.

[50] Romerius P., Ståhl O., Moëll C., Relander T., Cavallin-Ståhl E., Wiebe T., Giwercman Y.L., Giwercman A. (2011). High risk of azoospermia in men treated for childhood cancer. Int. J. Androl..

[51] Rosendahl M., Andersen C.Y., Ernst E., Westergaard L.G., Rasmussen P.E., Loft A., Andersen A.N. (2008). Ovarian function after removal of an entire ovary for cryopreservation of pieces of cortex prior to gonadotoxic treatment: A follow-up study. Hum. Reprod..

[52] Roy Moulik N., Keerthivasagam S., Pandey A., Agiwale J., Hegde K., Chatterjee G., Dhamne C., Prasad M., Chichra A., Srinivasan S. (2024). Treatment and follow-up of children with chronic myeloid leukaemia in chronic phase (CML-CP) in the tyrosine kinase inhibitor (TKI) era-Two decades of experience from the Tata Memorial Hospital paediatric CML (pCML) cohort. Br. J. Haematol..

[53] Schmidt K.L., Andersen C.Y., Loft A., Byskov A.G., Ernst E., Andersen A.N. (2005). Follow-up of ovarian function post-chemotherapy following ovarian cryopreservation and transplantation. Hum. Reprod..

[54] Shimizu M., Sawada A., Yamada K., Kondo O., Koyama-Sato M., Shimizu S., Komura H., Yasui M., Inoue M., Kawa K. (2012). Encouraging results of preserving ovarian function after allo-HSCT with RIC. Bone Marrow Transplant..

[55] Wang Y.L., Zhai Q.J., Wang Z.H., Yang X., Wang J.L., Zhu H.L. (2024). A retrospective study of ovarian tissue cryopreservation in female patients with hematological diseases for fertility preservation. Arch. Gynecol. Obstet..

[56] Wilhelmsson M., Vatanen A., Borgström B., Gustafsson B., Taskinen M., Saarinen-Pihkala U.M., Winiarski J., Jahnukainen K. (2014). Adult testicular volume predicts spermatogenetic recovery after allogeneic HSCT in childhood and adolescence. Pediatr. Blood Cancer.

[57] Zaletel L.Z., Bratanic N., Jereb B. (2004). Gonadal function in patients treated for leukemia in childhood. Leuk. Lymphoma.

[58] World Health Organization (2023). Infertility Prevalence Estimates: 1990–2021.

[59] Panasiuk A., Nussey S., Veys P., Amrolia P., Rao K., Krawczuk-Rybak M., Leiper A. (2015). Gonadal function and fertility after stem cell transplantation in childhood: Comparison of a reduced intensity conditioning regimen containing melphalan with a myeloablative regimen containing busulfan. Br. J. Haematol..

[60] Teinturier C., Hartmann O., Valteau-Couanet D., Benhamou E., Bougneres P. (1998). Ovarian function after autologous bone marrow transplantation in childhood: High-dose busulfan is a major cause of ovarian failure. Bone Marrow Transplant..

[61] Güemes M., Martín-Rivada Á., Arribas M.B., Andrés-Esteban E.M., Angulo B.M., Román J.P., Argente J. (2022). Endocrine Sequelae in 157 Pediatric Survivors of Hematopoietic Stem Cell Transplantation (HSCT). J. Endocr. Soc..

[62] Bresters D., Emons J.A., Nuri N., Ball L.M., Kollen W.J., Hannema S.E., Bakker-Steeneveld J.D., van der Bom J.G., Oostdijk W. (2014). Ovarian insufficiency and pubertal development after hematopoietic stem cell transplantation in childhood. Pediatr. Blood Cancer.

[63] Anderson R.A., Amant F., Braat D., D’Angelo A., Lopes S.M.C.D.S., Demeestere I., Dwek S., Frith L., Lambertini M., The ESHRE Guideline Group on Female Fertility Preservation (2020). ESHRE guideline: Female fertility preservation†. Hum. Reprod. Open.

[64] Balcerek M., Barnbrock A., Behringer K., Borgmann-Staudt A., Bürkle C., Dittrich R., Fehm T., Fey M., Germeyer A., Goeckenjan M., von Wolff M., Nawroth F. (2020). Indikation und Durchführung fertilitätsprotektiver Maßnahmen bei onkologischen und nicht-onkologischen Erkrankungen.

[65] Chevillon F., Clappier E., Arfeuille C., Cayuela J.-M., Dalle J.H., Kim R., Caye-Eude A., Chalas C., Abdo C., Drouineaud V. (2021). Minimal residual disease quantification in ovarian tissue collected from patients in complete remission of acute leukemia. Blood.

[66] Sönmezer M., Şükür Y.E., Saçıntı K.G., Özkavukçu S., Kankaya D., Atabekoğlu C.S., Seval G.C., Oktay K.H. (2023). Safety of ovarian cryopreservation and transplantation in patients with acute leukemia: A case series. Am. J. Obstet. Gynecol..

[67] Lotz L., Bender-Liebenthron J., Dittrich R., Häberle L., Beckmann M.W., Germeyer A., Korell M., Sänger N., Kruessel J.S., von Wolff M. (2022). Determinants of transplantation success with cryopreserved ovarian tissue: Data from 196 women of the FertiPROTEKT network. Hum. Reprod..

[68] Cortvrindt R., Smitz J., Van Steirteghem A. (1996). Ovary and ovulation: In-vitro maturation, fertilization and embryo development of immature oocytes from early preantral follicles from prepuberal mice in a simplified culture system. Hum. Reprod..

[69] Laronda M.M., Rutz A.L., Xiao S., Whelan K.A., Duncan F.E., Roth E.W., Woodruff T.K., Shah R.N. (2017). A bioprosthetic ovary created using 3D printed microporous scaffolds restores ovarian function in sterilized mice. Nat. Commun..

[70] von Wolff M., Bruckner T., Strowitzki T., Germeyer A. (2018). Fertility preservation: Ovarian response to freeze oocytes is not affected by different malignant diseases—An analysis of 992 stimulations. J. Assist. Reprod. Genet..

[71] Pryzant R.M., Meistrich M.L., Wilson G., Brown B., McLaughlin P. (1993). Long-term reduction in sperm count after chemotherapy with and without radiation therapy for non-Hodgkin’s lymphomas. J. Clin. Oncol..

[72] Ogouma L., Berthaut I., Lévy R., Hamid R.H., Prades M., Audouin M., Sermondade N., Dupont C. (2022). Testicular sperm extraction (TESE) outcomes in the context of malignant disease: A systematic review. Asian J. Androl..

[73] Skinner R., Mulder R.L., Kremer L.C., Hudson M.M., Constine L.S., Bardi E., Boekhout A., Borgmann-Staudt A., Brown M.C., Cohn R. (2017). Recommendations for gonadotoxicity surveillance in male childhood, adolescent, and young adult cancer survivors: A report from the International Late Effects of Childhood Cancer Guideline Harmonization Group in collaboration with the PanCareSurFup Consortium. Lancet Oncol..

[74] Mitchell R.T., Eguizabal C., Goossens E., Grynberg M., Jahnukainen K., Le Clef N., Mulder C.L., Neuhaus N., Rimmer M.P., ESHRE FP for Boys Working, Group. (2025). ESHRE good practice recommendations on fertility preservation involving testicular tissue cryopreservation in children receiving gonadotoxic therapies. Hum. Reprod..

[75] Anderson R.A., Cameron D., Clatot F., Demeestere I., Lambertini M., Nelson S.M., Peccatori F. (2022). Anti-Müllerian hormone as a marker of ovarian reserve and premature ovarian insufficiency in children and women with cancer: A systematic review. Hum. Reprod. Updat..

