# Supplementary material for: Fertility Outcomes in Leukemia Survivors by Treatment Modality: A Systematic Review and Meta-Analysis
Source: Cancers (Basel). 2026 Jul 30;18(15):2455. doi: 10.3390/cancers18152455 (PMC13464389; doi:10.3390/cancers18152455)
Supplement: Supplementary file 1 [file cancers-18-02455-s001.zip › cancers-4398674-supplementary.pdf]

Supplementary Materials

Supplementary text 1: search strategy

Research question: How do cancer therapies affect ovarian function and fertility in patients with leukemia?

Detailed search strategy

To identify potentially relevant publications on the topic, a search strategy was designed, and investigated in MEDLINE, Embase, and Cochrane Library. The core concepts of the search strategy consisted of 1. Leukemia, 2. Cancer therapies (chemotherapy or radiotherapy or immunotherapy or stem cell therapy or survivors of Leukemia), and 3. ovarian function/fertility. A medical information specialist developed an initial search strategy in Embase and tested it against a list of core references to ensure key publications were included. After refinement, the information specialist set up the search strategy for each information source based on database-specific index terms and free text. The free text search included synonyms, acronyms, and similar terms. In all databases in-built publication year filter was applied to limit the results for the period from 2000 to present. Additionally, in Cochrane the results were filtered by trials and systematic reviews. No other database-provided limits have been applied in any sources considering study types, languages or any other formal criteria. Studies concerning exclusively animals were excluded from the searches by using a double-negative search strategy based on the "Humans only" filters by Ovid. The search was finalized on 13/06/2024. The results were deduplicated in EndNote using the "Bramer method" . Results were imported into Covidence.

To ensure the inclusion of recent landmark studies published after our initial systematic search (June 2024), a supplementary literature update was conducted on June 29, 2026 during the peer review process. An AI-assisted search approach was employed using [Claude.ai – Opus 4.8 strong with connectors for PubMed, Clinicaltrials.gov, Consensus, Scite, Elicit, bioRxiv/medRxiv]. Identified records were screened using the original eligibility criteria. Newly identified eligible studies were integrated into the synthesis and meta-analysis. This targeted update is documented in the PRISMA flow diagram under 'Identification of studies via other methods'."

Table S1. Search Strategy.

Search date	Database searched	Platform	Dates of coverage	Records	Deduplicated records
13 June 2024	Embase	Ovid	1974 – 12 June 2024	4616	3354
13 June 2024	Medline	Ovid	1946 - 12 June 2024	1409	1408
13 June 2024	Cochrane Database of Systematic Reviews	Wiley	1992 - present	1	0
13 June 2024	Cochrane Central Register of Controlled Trials	Wiley	1992 - present	156	103

	Total references	6182	4866

Search strategies

Embase <1974 to 2024 June 12>
Search date: 13 June 2024

1 exp leukemia/ 383626

2 (AML or refractory anemia with excess blasts in transformation or myeloid sarcoma* or Acute panmyelos* or Transient abnormal myelopoies* or chronic myeloleu#?emia* or blast cell cris#s or CML or cmml or lymphoblast* lymphom* or childhood ALL or leu#?emia* or jmml).ti,ab,kw. 436347

3 or/1-2 529826

4 exp antineoplastic agent/ or exp multimodality cancer therapy/ or exp cancer chemotherapy/ or exp antineoplastic protocol/ 3266053

5 (chemotherap* or chemo-therap* or chemoradiotherap* or chemo-radiotherap* or adjuvant drug therap* or carcinochemotherap* carcino-chemotherap* or antineoplastic agent* or anti-neoplastic agent* or antineoplastic drug* or anti-neoplastic drug* or antitumo?r agent* or anti-tumo?r agent* or antitumo?r drug* or anti-tumo?r drug* or anticancer* agent* or anti-cancer* agent* or anticancer* drug* or anti-cancer drug* or anticarcinogen* or anti-carcinogen* or anticancerogen* or anti-cancerogen* or ((cancer* or tumo?r* or neoplas*) adj3 treat*)).ti,ab,kw. 1378425

6 exp cancer radiotherapy/ 347015

7 (Radio-therap* or radiotherap* or radiationtherap* or chemoradiotherap* or radiochemotherap* or protontherap* or radiosurg* or radio-surg* or irradiation* or x-ray-therap* or therap* radiolog* or IMRT* or IORT* or radioimmunotherap* or radio-immuno-therap* or ((radiat* or irradiat* or radioisotope* or radio-isotope* or chemoradio or chemo-radio or radiochemo or radio-chemo or proton or x-ray or xray) adj2 (therap* or oncolog* or brachytherap* or brachy-therap*))).ti,ab,kw. 742476

8 3 and surviv*.ti,ab,kw. 116249

9 exp bone marrow transplantation/ or exp stem cell transplantation/ 262495

10 (((bone marrow* or h?ematopoiet* or stem cell* or hsc) adj4 (transplant* or transfer* or graft* or transfus* or infus* or therap*)) or hsct).ti,ab,kw. 249678

11 exp immunotherapy/ 338691

12 (biologic* response modifier* therap* or BRM? Therap* or immun* therap* or imun* therap* or immun* treat* or imun* treat* or immun* intervent* or imun* intervent* or immunotherap* or imunotherap* or immunity modulator* or antibod* therap*).ti,ab,kw. 333455

13 or/4-12 4625275

14 exp fertility/ or exp infertility/ or exp semen analysis/ or exp gonad/ or exp amenorrhea/ or exp early menopause/ or reproduction/ or gametogenesis/ or spermatozoon/ or exp menarche/ or exp estrogen therapy/ or exp inhibin B/ or exp testis size/ 630976

15 (fertili#ation* or fertility or fertile or fecund* or subfecund* or sub-fecund* or infecund* or infertility or (ovar* adj4 sterility) or subfert* or sub-fert* or anovularit* or gonad* or reproductive organ* or reproduction* or gamete-producing gland* or ovarian reserve* or ovary or ovaries or ovarian follicle* or oogenes#s or oocyte* or amenorrhea* or premature menopaus* or early menopaus* or climacterium pr?ecox or Gonadotropin* or AMH or Anti-Mu?llerian Hormone* or Antimu?llerian Hormone* or Anti-Mu?llerian Factor* or Mu?llerian Inhibiting Hormone* or mu?llerian inhibitor* or FSH or Follicle

Stimulating Hormone* or Folliculostimulating Hormone* or Follitropin or FSH-releasing hormone* or LH-FSH or testis or testes or testicle* or spermatogenes* or sperm* or semen or gametogenes* or hypogonadism* or hypo-gonadism* or "reproductive system*" or azoospermia* or spermatozoon* or ((resumpt* or recover*) adj3 menstruat*) or menarch* or "menstruat* onset*" or "first menstruat*" or "puberal h?emorrhage*" or (?estrogen* adj3 (substitut* or administrat* or replace* or therap*)) or "hormon* replacement therap*" or hrt or inhibin b or inhibin subunit b or (follicular matur* or ovul* or oligomenorrhoe* or folliculo* or oncofertil*)).ti,ab,kw. 967460

16 or/14-15 1129966

17 3 and 13 and 16 6131

18 (exp animal/ or exp invertebrate/ or nonhuman/ or animal experiment/ or animal tissue/ or animal model/ or exp plant/ or exp fungus/ or (rat? or mouse or mice or animal?).ti.) not (exp human/ or human tissue/) 8003328

19 17 not 18 5547

20 limit 19 to yr="2000 -Current" 4616

https://ovidsp.ovid.com/ovid-web.cgi?T=JS&NEWS=N&PAGE=main&SHAREDSEARCHID=3UIxc6Dy5ow674j9mmH9TxB5QYEVgJAezBvRQI9QSxG8qWL8wTHltGvxQK1r55pJU

****

Ovid MEDLINE(R) ALL <1946 to June 12, 2024>
Search date: 13 June 2024

1 exp Leukemia/ 258749

2 (AML or refractory anemia with excess blasts in transformation or myeloid sarcoma* or Acute panmyelos* or Transient abnormal myelopoies* or chronic myeloleu#?emia* or blast cell cris#s or CML or cmml or lymphoblast* lymphom* or childhood ALL or leu#?emia* or jmml).ti,ab,kw. 308100

3 or/1-2 367943

4 exp Antineoplastic Agents/ or exp Combined Modality Therapy/ or exp Antineoplastic Combined Chemotherapy Protocols/ or exp chemotherapy, adjuvant/ or exp Antineoplastic Protocols/ or exp chemoradiotherapy/ or radioimmunotherapy/ or exp radiotherapy, adjuvant/ 1527232

5 (chemotherap* or chemo-therap* or chemoradiotherap* or chemo-radiotherap* or adjuvant drug therap* or carcinochemotherap* carcino-chemotherap* or antineoplastic agent* or anti-neoplastic agent* or antineoplastic drug* or anti-neoplastic drug* or antitumo?r agent* or anti-tumo?r agent* or antitumo?r drug* or anti-tumo?r drug* or anticancer* agent* or anti-cancer* agent* or anticancer* drug* or anti-cancer drug* or anticarcinogen* or anti-carcinogen* or anticancerogen* or anti-cancerogen* or ((cancer* or tumo?r* or neoplas*) adj3 treat*)).ti,ab,kw. 913272

6 exp Radiotherapy/ 212016

7 (Radio-therap* or radiotherap* or radiationtherap* or chemoradiotherap* or radiochemotherap* or protontherap* or radiosurg* or radio-surg* or irradiation* or x-ray-therap* or therap* radiolog* or IMRT* or IORT* or radioimmunotherap* or radio-immuno-therap* or ((radiat* or irradiat* or radioisotope* or radio-isotope* or chemoradio or chemo-radio or radiochemo or radio-chemo or proton or x-ray or xray) adj2 (therap* or oncolog* or brachytherap* or brachy-therap*))).ti,ab,kw. 536751

8 3 and surviv*.ti,ab,kw. 55459

9 exp Bone Marrow Transplantation/ or exp Stem Cell Transplantation/ 142662

10 (((bone marrow* or h?ematopoiet* or stem cell* or hsc) adj4 (transplant* or transfer* or graft* or transfus* or infus* or therap*)) or hsct).ti,ab,kw. 146742

11 exp Immunotherapy/ 346731

12 (biologic* response modifier* therap* or BRM? Therap* or immun* therap* or imun* therap* or immun* treat* or imun* treat* or immun* intervent* or imun* intervent* or immunotherap* or imunotherap* or immunity modulator* or antibod* therap*).ti,ab,kw. 214250

13 or/4-12 2896469

14 exp Fertility/ or exp Infertility/ or exp Gonads/ or Amenorrhea/ or anovulation/ or menopause, premature/ or Reproduction/ or Gametogenesis/ or Spermatozoa/ or exp Menarche/ or exp Estrogen Replacement Therapy/ or exp Inhibin-beta Subunits/405521

15 (fertili#ation* or fertility or fertile or fecund* or subfecund* or sub-fecund* or infecund* or infertility or (ovar* adj4 sterility) or subfert* or sub-fert* or anovularit* or gonad* or reproductive organ* or reproduction* or gamete-producing gland* or ovarian reserve* or ovary or ovaries or ovarian follicle* or oogenes#s or oocyte* or amenorrhea* or premature menopaus* or early menopaus* or climacterium pr?ecox or Gonadotropin* or AMH or Anti-Mu?llerian Hormone* or Antimu?llerian Hormone* or Anti-Mu?llerian Factor* or Mu?llerian Inhibiting Hormone* or mu?llerian inhibitor* or FSH or Follicle Stimulating Hormone* or Folliculostimulating Hormone* or Follitropin or FSH-releasing hormone* or LH-FSH or testis or testes or testicle* or spermatogenes* or sperm* or semen or gametogenes* or hypogonadism* or hypo-gonadism* or "reproductive system*" or azo-ospermia* or spermatozoon* or ((resumpt* or recover*) adj3 menstruat*) or menarch* or "menstruat* onset*" or "first menstruat*" or "puberal h?emorrhage*" or (?estrogen* adj3 (substitut* or administrat* or replace* or therap*)) or "hormon* replacement therap*" or hrt or inhibin b or inhibin subunit b or (follicular matur* or ovul* or oligomenorrhoe* or folliculo* or oncofertil*)).ti,ab,kw. 821200

16 or/14-15 927776

17 3 and 13 and 16 2692

18 (exp animal/ or exp invertebrate/ or animal experiment/ or animal model/ or exp plant/ or exp fungus/) not exp human/ 5671858

19 17 not 18 2304

20 limit 19 to yr="2000 -Current" 1409

https://ovidsp.ovid.com/ovid-web.cgi?T=JS&NEWS=N&PAGE=main&SHAREDSEARCHID=4WXZ8rcPewJnSZWZTjrFnAIH1nk0aZbcYAcnWwcvcCpm7z9x6ezTP2Trpwev4LCWZ

****

Cochrane Database
Search date: 13 June 2024

#1 [mh "leukemia"] 6576

#2 (AML or (refractory NEXT an*emia NEXT "with excess blasts in transformation") or (myeloid NEXT sarcoma*) or (Acute NEXT panmyelos*) or ("Transient abnormal" NEXT myelopoies*) or (chronic NEXT myeloleu*emia*) or ("blast cell" NEXT cris?s) or CML or cmml or (lymphoblast* NEXT lymphom*) or "childhood ALL" or leu*emia* or jmml):ti,ab,kw 18933

#3 #1 OR #2 18934

#4 [mh "Antineoplastic Agents"] OR [mh "Combined Modality Therapy"] OR [mh "Antineoplastic Combined Chemotherapy Protocols"] OR [mh "chemotherapy, adjuvant"] OR [mh "Antineoplastic Protocols"] OR [mh "Chemoradiotherapy"] OR [mh ^Radioimmunotherapy] OR [mh "Radiotherapy, Adjuvant"] 58708

#5 (chemotherap* or chemo-therap* or chemoradiotherap* or chemo-radiotherap* or (adjuvant NEXT drug NEXT therap)* or carcinochemotherap* or carcino-chemotherap* or (antineoplastic NEXT agent*) or (anti-neoplastic NEXT agent*) or (antineoplastic NEXT drug*) or (anti-neoplastic NEXT drug*) or (antitumo?r NEXT agent*) or (anti-tumo?r NEXT agent*) or (antitumo?r NEXT drug*) or (anti-tumo?r NEXT drug*) or (anticancer* NEXT agent*) or (anti-cancer* NEXT agent*) or (anticancer* NEXT drug*) or (anti-cancer NEXT drug*) or anticarcinogen* or anti-carcinogen* or anticancerogen* or anti-cancerogen* or (cancer* NEXT treat*) or (tumo?r* NEXT treat*) or (neoplas* NEXT treat*)):ti,ab 11480

#6 [mh Radiotherapy] 10030

#7 (Radio-therap* or radiotherap* or radiationtherap* or (radiation NEXT therap*) or (irradiation NEXT therap*) or (oncologic* NEXT radiation*) or (oncologic* NEXT irradiation*) or radiochemotherap* or radio-chemo-therap* or (radiochemo* NEXT therap*) or (radio-chemo* NEXT therap*) or brachytherapy* or brachy-therap* or (radioisotope* NEXT therap*) or (radio-isotope* NEXT therap*) or (chemo-radio NEXT therap*) or (radiochemo NEXT therap*) or (radio-chemo NEXT therap*) or (proton* NEXT therap*) or protontherap* or (x-ray* NEXT therap*) or (xray* NEXT therap*) or radiosurg* or radio-surg* or irradiation* or x-ray-therap* or (therap* NEXT radiolog*) or IMRT* or IORT* or radioimmunotherap* or radio-immuno-therap*):ti,ab 44206

#8 #3 AND surviv*:ti,ab,kw 8658

#9 [mh "Bone Marrow Transplantation"] OR [mh "Stem Cell Transplantation"] 4908

#10 ((Bone NEXT marrow* NEXT transplant*) or (bone NEXT marrow* NEXT cell NEXT transfer*) or (bone NEXT marrow* NEXT graft*) or (bone NEXT marrow* NEXT transfus*) or (bone NEXT marrow* NEXT infus*) or (bone NEXT marrow* NEXT therap*) or (bone NEXT marrow* NEXT purg*) or (stem NEXT cell* NEXT transplant*) or (stem NEXT cell* NEXT therap*) or (stem NEXT cell* NEXT based NEXT therap*) or h?ematopoiet* or hsc):ti,ab 13633

#11 [mh Immunotherapy] 12137

#12 ((biologic* NEXT response NEXT modifier* NEXT therap*) or (BRM? NEXT Therap*) or (immun* NEXT therap*) or (imun* NEXT therap*) or (immun* NEXT treat*) or (imun* NEXT treat*) or (immun* NEXT intervent*) or (imun* NEXT intervent*) or immunotherap* or imunotherap* or (immunity NEXT modulator*) or (antibod* NEXT therap*)):ti,ab 16373

#13 #4 OR #5 OR #6 OR #7 OR #8 OR #9 OR #10 OR #11 OR #12 139165

#14 [mh Fertility] OR [mh Infertility] OR [mh Gonads] OR [mh ^Amenorrhea] OR [mh ^anovulation] OR [mh ^"menopause, premature"] OR [mh ^Reproduction] OR [mh ^Gametogenesis] OR [mh ^Spermatozoa] OR [mh Menarche] OR [mh "Estrogen Replacement Therapy"] OR [mh "Inhibin-beta Subunits"] 9966

#15 (fertili*ation* OR fertility OR fertile OR fecund* OR subfecund* OR sub-fecund* OR infecund* OR infertility OR ovari* OR subfert* OR sub-fert* OR anovularit* OR gonad* OR (reproductive NEXT organ*) OR reproduction* OR (gamete-producing NEXT gland*) OR ovary OR oogenesis OR oocyte* OR amenorrhea* OR (premature NEXT menopaus*) OR (early NEXT menopaus*) OR (climacterium NEXT pr*ecox) OR Gonadotropin* OR AMH OR (Anti-Mu*llerian NEXT Hormone*) OR (Antimu*llerian NEXT Hormone*) OR (Anti-Mu*llerian NEXT Factor*) OR (Mu*llerian NEXT Inhibiting NEXT Hormone*) OR (mu*llerian NEXT inhibitor*) OR FSH OR (Follicle NEXT

Stimulating NEXT Hormone*) OR (Folliculostimulating NEXT hormone*) OR Follitropin OR (FSH-releasing hormone*) OR LH-FSH OR testis OR testes OR testicle* OR spermatogenes* OR sperm* OR semen OR gametogenes* OR hypogonadism* OR hypo-gonadism* OR (reproductive NEXT system*) OR azoospermia* OR spermatozoon* OR ((resumpt* OR recover*) NEAR/3 menstruat*) OR menarch* OR (menstruat* NEXT onset*) OR (first NEXT menstruat*) OR (puberal NEXT h*emorrhage*) OR (*estrogen* NEAR/3 (substitut* OR administrat* OR replace* OR therap*)) OR (hormon* NEXT replacement NEXT therap*) OR hrt OR (inhibin NEXT b) OR (inhibin NEXT subunit NEXT b) OR oncofertil*):ti,ab 52066

#16 #14 OR #15 53774

#17 #3 AND #13 AND #16 with Cochrane Library publication date Between Jan 2000 and Jun 2024, in Cochrane Reviews, Trials 157

Table S2. PRISMA 2020 checklist.

Section and Topic	Item #	Checklist item	Location where item is reported
**TITLE**			
Title	1	Identify the report as a systematic review.	Title
**ABSTRACT**			
Abstract	2	See the PRISMA 2020 for Abstracts checklist.	yes
**INTRODUCTION**			
Rationale	3	Describe the rationale for the review in the context of existing knowledge.	introduction
Objectives	4	Provide an explicit statement of the objective(s) or question(s) the review addresses.	introduction
**METHODS**			
Eligibility criteria	5	Specify the inclusion and exclusion criteria for the review and how studies were grouped for the syntheses.	methods section
Information sources	6	Specify all databases, registers, websites, organisations, reference lists and other sources searched or consulted to identify studies. Specify the date when each source was last searched or consulted.	methods section
Search strategy	7	Present the full search strategies for all databases, registers and websites, including any filters and limits used.	supplementary material
Selection process	8	Specify the methods used to decide whether a study met the inclusion criteria of the review, including how many reviewers screened each record and each report retrieved, whether they worked independently, and if applicable, details of automation tools used in the process.	methods section
Data collection process	9	Specify the methods used to collect data from reports, including how many reviewers collected data from each report, whether they worked independently, any processes for obtaining or confirming data from study investigators, and if applicable, details of automation tools used in the process.	methods section
Data items	10a	List and define all outcomes for which data were sought. Specify whether all results that were compatible with each outcome domain in each study were sought (e.g. for all measures, time points, analyses), and if not, the methods used to decide which results to collect.	methods section
	10b	List and define all other variables for which data were sought (e.g. participant and intervention characteristics, funding sources). Describe any assumptions made about any missing or unclear information.	methods section

Section and Topic	Item #	Checklist item	Location where item is reported
Study risk of bias assessment	11	Specify the methods used to assess risk of bias in the included studies, including details of the tool(s) used, how many reviewers assessed each study and whether they worked independently, and if applicable, details of automation tools used in the process.	methods section
Effect measures	12	Specify for each outcome the effect measure(s) (e.g. risk ratio, mean difference) used in the synthesis or presentation of results.	methods section
Synthesis methods	13a	Describe the processes used to decide which studies were eligible for each synthesis (e.g. tabulating the study intervention characteristics and comparing against the planned groups for each synthesis (item #5)).	methods section
	13b	Describe any methods required to prepare the data for presentation or synthesis, such as handling of missing summary statistics, or data conversions.	methods section
	13c	Describe any methods used to tabulate or visually display results of individual studies and syntheses.	methods section
	13d	Describe any methods used to synthesize results and provide a rationale for the choice(s). If meta-analysis was performed, describe the model(s), method(s) to identify the presence and extent of statistical heterogeneity, and software package(s) used.	methods section
	13e	Describe any methods used to explore possible causes of heterogeneity among study results (e.g. subgroup analysis, meta-regression).	methods section
	13f	Describe any sensitivity analyses conducted to assess robustness of the synthesized results.	methods section
Reporting bias assessment	14	Describe any methods used to assess risk of bias due to missing results in a synthesis (arising from reporting biases).	methods section
Certainty assessment	15	Describe any methods used to assess certainty (or confidence) in the body of evidence for an outcome.	methods section and quality assessment
**RESULTS**			
Study selection	16a	Describe the results of the search and selection process, from the number of records identified in the search to the number of studies included in the review, ideally using a flow diagram.	flow diagram
	16b	Cite studies that might appear to meet the inclusion criteria, but which were excluded, and explain why they were excluded.	flow diagram
Study characteristics	17	Cite each included study and present its characteristics.	quality assessment and results section
Risk of bias in studies	18	Present assessments of risk of bias for each included study.	quality assessment
Results of individual studies	19	For all outcomes, present, for each study: (a) summary statistics for each group (where appropriate) and (b) an effect estimate and its precision (e.g. confidence/credible interval), ideally using structured tables or plots.	results section

Section and Topic	Item #	Checklist item	Location where item is reported
Results of syntheses	20a	For each synthesis, briefly summarise the characteristics and risk of bias among contributing studies.	quality assessment and results section
	20b	Present results of all statistical syntheses conducted. If meta-analysis was done, present for each the summary estimate and its precision (e.g. confidence/credible interval) and measures of statistical heterogeneity. If comparing groups, describe the direction of the effect.	results section and supplementary materials
	20c	Present results of all investigations of possible causes of heterogeneity among study results.	results section and supplementary materials
	20d	Present results of all sensitivity analyses conducted to assess the robustness of the synthesized results.	results section and supplementary materials
Reporting biases	21	Present assessments of risk of bias due to missing results (arising from reporting biases) for each synthesis assessed.	results section and supplementary materials
Certainty of evidence	22	Present assessments of certainty (or confidence) in the body of evidence for each outcome assessed.	results section and supplementary materials and quality assessment
**DISCUSSION**			
Discussion	23a	Provide a general interpretation of the results in the context of other evidence.	discussion
	23b	Discuss any limitations of the evidence included in the review.	subsection limitations
	23c	Discuss any limitations of the review processes used.	discussion

Section and Topic	Item #	Checklist item	Location where item is reported
	23d	Discuss implications of the results for practice, policy, and future research.	discussion
**OTHER INFORMATION**			
Registration and protocol	24a	Provide registration information for the review, including register name and registration number, or state that the review was not registered.	PROSPERO registration number was given
	24b	Indicate where the review protocol can be accessed, or state that a protocol was not prepared.	yes
	24c	Describe and explain any amendments to information provided at registration or in the protocol.	Not applicable
Support	25	Describe sources of financial or non-financial support for the review, and the role of the funders or sponsors in the review.	yes
Competing interests	26	Declare any competing interests of review authors.	yes
Availability of data, code and other materials	27	Report which of the following are publicly available and where they can be found: template data collection forms; data extracted from included studies; data used for all analyses; analytic code; any other materials used in the review.	Data can be accessed after reasonable request with the authors

Table S3. Quality assessment of included studies.

First author, year of publication	Selection				Comparability<br><br>cohort comparability based on the design or analysis controlled for confounders		Outcome			Total	Quality assess ment	Comments
	Representativeness of exposed cohort	Selection of non-exposed cohort	Ascertainment of exposure (secure records)	Demonstration that outcome of interest was not present at study start	a) study controls for main factor (e.g. age, sex)	b) study controls for additional factors (e.g. ethnicity, comorbidities)	Assessment of outcome (independent blind assessment)	Sufficient length of follow-up for outcomes to occur	Adequacy of follow-up of cohorts (≥ 80% remained untill outcome)			

Abir et al. (2016)	*	-	*	*	-	-	*	*	-	5/9	poor	no control group
Afify et al. (2002)	*	-	*	*	-	-	*	*	-	5/9	poor	no control group
Akisada et al. (2023)	*	-	*	-	-	-	*	*	-	4/9	poor	no control group
Ashizawa et al. (2019)	*	-	*	*	-	-	*	-	-	4/9	poor	no control group
Assouline et al. (2013)	*	-	*	*	-	-	*	-	*	5/9	poor	no control group
Bahadur et al. (2005)	*	-	*	*	-	-	*	-	-	4/9	poor	no control group
Balcerek et al. (2012a)	*	-	*	-	-	-	*	*	-	4/9	poor	no control group
Balcerek et al. (2012b)	*	-	*	-	-	-	-	*	-	3/9	poor	no control group
Bath et al. (2001)	*	-	*	-	*	-	*	*	-	5/9	fair	control group: n=16, controls for age
Bhatt et al. (2021)	*	*	*	-	*	*	*	*	-	7/9	good	community control group: n=450, controls for age, sex, race
Biasin et al. (2015)	*	-	*	-	-	-	*	*	-	4/9	poor	no control group
Byrne et al. (2004), female cohort	*	*	*	-	*	*	-	-	*	6/9	poor	community control group: n=170, female siblings
Byrne et al. (2004), male cohort	*	*	*	-	*	*	-	-	*	6/9	poor	community control group: n=145, male siblings
Chabut et al. (2023)	*	-	*	*	-	-	*	*	*	6/9	poor	no control group

Courbiere et al. (2023)	*	-	*	*	-	-	*	*	-	5/9	poor	no control group for relevant parameter
Couto-Silva et al. (2001)	*	-	*	*	-	-	*	*	*	6/9	poor	no control group
Even-Or et al. (2016)	*	-	*	-	-	-	*	*	-	4/9	poor	no control group
Forgeard et al. (2020)	*	-	*	-	-	-	*	*	-	4/9	poor	no control group
Freycon et al. (2019)	*	-	*	-	-	-	*	*	*	5/9	poor	no control group
Frisk et al. (2004)	*	-	*	*	-	-	*	*	*	6/9	poor	no control group
Fujino et al. (2019)	*	-	*	-	-	-	*	*	*	5/9	poor	no control group
Gozdasoglu et al. (2002)	*	-	*	-	-	-	*	*	*	5/9	poor	no control group
Green et al (2017)	*	-	*	-	-	-	*	*	-	4/9	poor	no control group
Jadoul et al.(2017)	*	-	*	-	-	-	-	*	-	3/9	poor	no control group
Jensen et al (2016)	*	-	*	-	-	-	*	*	-	4/9	poor	no control group
Komori et al. (2019)	*	-	*	-	-	-	*	*	-	4/9	poor	no control group
Letourneau et al. (2012)	*	-	*	-	-	-	-	*	-	3/9	poor	no control group
Leung et al. (2000)	*	-	*	-	-	-	*	*	-	4/9	poor	no control group
Maeda et al. (2003)	*	-	*	-	-	-	*	*	-	4/9	poor	no control group
Mancini et al. (2008)	*	-	*	-	-	-	*	*	-	4/9	poor	no control group
Marquis et al. (2010)	*	-	*	-	-	-	*	*	-	4/9	poor	80 much older, healthy controls age at sperm analysis: 35 (range: 18–51)

Naessen et al. (2014)	*	-	*	*	-	-	*	*	*	6/9	poor	no control group
Nesr et al. (2024)	*	-	*	*	-	-	*	*	-	5/9	poor	no control group
Netterlid et al. (2021)	*	*	*	-	*	*	*	*	-	7/9	good	164 controls, matched for age, sex, ethnicity, residence, and smoking habits
Poganitsch-Korhonen et al (2017)	*	-	*	-	-	-	*	*	-	4/9	poor	no control group
Romerius et al (2010)	*	-	*	-	-	-	*	*	-	4/9	poor	no control group
Rosendahl et al (2008)	*	-	*	-	-	-	*	*	*	5/9	poor	no control group
RoyMoulik el al. (2024)	*	-	*	-	-	-	*	*	-	4/9	poor	no control group
Schmidt et al. (2005)	*	-	*	-	-	-	*	*	-	4/9	poor	no control group
Shimizu et al. (2012)	*	-	*	*	-	-	*	*	-	4/9	poor	no control group
Wang et al. (2024)	*	-	*	*	-	-	*	-	-	4/9	poor	no control group
Wilhelmsson et al. (2014)	*	-	*	-	-	-	*	*	-	4/9	poor	no control group
Zaletel et al. (2004)	*	-	*	-	-	-	*	-	-	3/9	poor	no control group

Newcastle-Ottawa Quality Assessment Form for Cohort Studies. solely retrospective studies did not receive a star in the category „adequacy of follow-up of cohorts“.

Table S4. Characteristics of included studies.

**First author (year of publication)**	**Country**	**Study design**	**Participants (n)**	**diagnosis**	**Treatment without HSCT**	**CI**	**HSCT**	**TBI**	**TI**	**Treatment era**	**Age at diagnosis (mean/**	**Age at assessment (mean/median, range)**	**Follow-up period (mea**	**cases of infertility (n)**	**cases of subfertility (n)**	**Definition of infertility**	**Definition of subfertility**

											median, range) in years	in years	n/median, range) in years				
Abir et al. (2016)	Israel	prospective	4 (4 females)	AML (2); ALL (2)	chemo only (alkylating agents)	-	-	-	-	2007 - 2014	12.25 (range: 3-16)	12.25 (range: 3-16)	NA	2/4	-	AMH undetectable	-
Afify et al. (2002)	Australia	retrospective longitudinal	16 (6 females, 10 males)	AML (16)	-	-	16	-	1/16	1987-1995	10.9 (range: 1- 15)	14.57 (range: 6-22.6)	4.9 (range: 2-10)	6/16<br>females: 5/6<br>males: 1/10	-	HRT	-
Akisada et al. (2023)	Japan	retrospective	6 (3 female; 3 male)	ALL (6)	-	-	5	1	-	1996 - 2011	1-11 months	12 – 26	13	4/6	0/3	hypergonadotropic hypogonadism	FSH elevated but <25
Ashizawa et al. (2019)	Japan	retrospective + survey	11 (11 females)	ALL (5); AML (6)	-	-	11	11	-	2007-2018	23 (range: 19-33)	NA	3.79 (range: 0.2-10)	5/11	-	AMH <0.1	-
Assouline et al. (2013)	France	retrospective longitudinal	7 (7 females)	ALL (2); AML (5)	-	-	7	3 (TBI 2 Gy)	-	2000 - 2010	27 (range: 18-35)	32.5 (range 22-46)	4.5 (range: 0.4–22.8)	7/7	-	POI	-
Bahadur et al. (2005)	United Kingdom	retrospective longitudinal	13 (13 males)	acute leukemia	chemo only (no details given)	-	-	-	-	1976-2002	NA	30.4 (SD ±7.7)	3.1 (range: 0.25–15.2)	6/13	1/13	azoospermia	oligospermia
Balcerek et al. (2012)	Germany	a.) retrospective longitudinal<br>b.) survey	a.) 59 (26 females; 33 males)<br>b.) 551	acute leukemia	NA	NA	NA	NA	NA	2008-2009	7 (range: 0-15)	25 (range: 19-43)	~18	a.) 15/59<br>b.) 8/551	-	a.) hormone analysis	

			(551 females)													b.) permanent amenorrhea	
Bath et al. (2001)	United Kingdom	prospective	12 (12 females)	ALL	12	12	0	0	12	~ 1980-1990	4.7 (range: 1.9–13.1)	20.8 (range: 15.8–32.8)	16.1	0/12	0/12	regular menstrual cycle	oligomenorrhea
Bhatt et al. (2021)	USA	prospective	98 (74 females, 24 males)	AML	42 females; 3 males	HSCT group: ~4.5% chemo only group: ~14.9 %	32 females; 21 males	HSCT group: ~68 % chemo only group: 0	-	~1990-2000	HSCT group: 9.7 (SD ±5.8) chemo only group: 9.5 (SD ±6.2)	HSCT group: 30.4 (SD ±7.6) chemo only group: 33.6 (SD ±9.1)	HSCT group: 20.6 (SD ±5.4) chemo only group: 24.1 (SD ±9.3)	29/98 females: 14/74 males: 15/24	-	females: POI; males: azoospermia	-
Biasin et al. (2015)	Italy	retrospective longitudinal	18 (18 females)	AML ALL CML	-	-	18	majority TBI; rest BU	-	2000-2013	11.1 (range: 0–17.5)	18.6 (range: 5.5–29.4)	6.5 (range: 0.3–13.7)	17/18	-	amenorrhea (FSH >25 in all cases)	-
Byrne et al. (2004), female cohort	USA	survey	11 (11 females)	ALL	chemo + CI 22.8% received alkylating agents	11	-	-	-	treatment: 1970-1987 survey 1990 - 1991	10.7	22.6	at least 2 years	9/11	-	WHO definition: ≥ 12 months no pregnancy conceived	-
Byrne et al. (2004), male cohort	USA	survey	213 (213 male)	ALL	chemo + CI, 33.5% received	90.4% received cranial RT	-	-	-	treatment: 1970-1987	10.9	22.8	at least 2 years	64/213	-	WHO definition: ≥ 12 mont	-

					alkylating agents					survey 1990 - 1991						hs no pregnancy fathered	
Chabut et al. (2023)	France	prospective	178 (178 females)	ALL (108); AML (70)	-	-	178	116	-	2004-2020	7.7 (range 4.79 - 10.9)	24.9 (range 21.08 - 30.1)	18 (range: 14.2-23.3)	139/178	-	POI	-
Courbiere et al. (2023)	France	prospective	88 (88 females)	acute leukemia	34 received alkylating agents	-	88	54	-	~1990 - 2010	9.1 (SD ±0.3)	26.5 (SD ±6.3)	16.4 (SD ±0.5)	71/88	-	POI with or without HRT	-
Couto-Silva et al. (2001)	France	retrospective longitudinal	35 (13 females; 22 males)	ALL (23; 8 females; 15 males); AML (12; 5 females; 7 males)	-	-	35 (females: 13; males: 22)	33 (females: 13; males: 20)	-	~1990 - 2000	females: 7.2 (range: 0.6 - 13.1; SD ±0.6) males: 8.1 (range: 2-13.6; SD ± 0.5)	females: 14.3 (range: 10.1 - 19.7; SD ±0.4) males: 14.7 (range: 11-20.6; SD± 0.3)	females: 7.1 males: 6.6	13/35 females: 10/13 males: 3/22	5/35 females: 2/13 3/22 males	females + males: FSH > 25 IU/L	females + males :: FSH 10-25 IU/L
Even-Or et al. (2016)	Israel	retrospective longitudinal	7 (7 females)	AML (7)	-	-	7	-		1996 - 2006	10.5 (range: 1–19)	25.5 (range: 13–36)	16 (range: 9-20)	0/7	5/7	females: AMH undetectable	females: AMH <0.5
Forgeard et al. (2020)	France	retrospective longitudinal	16 (16 females)	acute leukemia	-	-	16	-	-	treatment~ 2006 study: 2014-2016	23.4 (range: 18.3 - 28)	30.9 (range: 24.2 - 36.9)	12.2	13/16	-	FSH > 25 IU/L, AMH undetectable	
Freycon et al. (2019)	France	retrospective longitudinal	14 (7 females; 7	mixed leukemia	-	-	14	14	NA	1988 - 2010	before the age of 15	25.0	14.8	7/7	7/7	males: azoospermia	females: AMH <0.5

			males)	(AML, ALL, CML)													
Frisk et al. (2004)	Sweden	retrospective longitudinal	17 (6 females; 11 males)	ALL (17)	-	7 (2 females, 5 males)	17	17	5/11 males	1985 - 1994	8.3 (range: 3.6–13.1)	19.5 (range: 15.9–22.1)	~11.2	13/17<br>females: 4/6<br>males: 9/11	-	females + males: HRT	-
Fujino et al. (2019)	Japan	retrospective	11 (11 females)	ALL (5); AML (6)	-	-	11 (melphalan-based reduced intensity conditioning (RIC))	TBI < 8 Gy: 2/11	-	2015	at diagnosis: 8 (range: 1–16)<br>at HSCT: 10 (range: 1–18)	17 (range: 12–25)	~7	1/11	1/11	FSH > 25 IU/L	FSH 10-25 IU/L
Gozdasoglu et al. (2002)	Turkey	retrospective longitudinal	16 (4 females; 12 males)	acute leukemia	16	13	-	-	5/6 males	~1990 -2000	7.96 (SD ±3.43)	17.38 (SD ±3.81)<br>males: 16.87 (SD ±4.03)<br>females: 18.89 (SD ±3.00)	5.20 (SD ±3.43)	8/16<br>females: 0/4<br>males: 8/12	1/9<br>females: 0/4<br>males: 1/5	females: amenorrhea<br>males: hypergonadotropic hypogonadism or azoospermia	females: oligomenorrhea<br>males: oligospermia
Green et al (2017)	USA	cross-sectional study	173 (173 males)	ALL (173)	173 alkylating agent	91/173	-	-	-	~1980 -2000	irradiated patients:	irradiated patients: 32.9 (SD ±7.8)	irradiated patients:	65/173<br>irradiated	46/173	azoospermia	oligospermia

					chemotherapy +/- cranial radiation therapy (CRT)	>0 to 20 Gy: 62 >20 to 26 Gy: 29					6.6 (SD ±4.4) non-irradiated patients: 7.5 (SD ±5.0)	non-irradiated patients: 26.2 (SD ±5.6)	26.3 (SD ±6.3) non-irradiated patients: 18.7 (SD ±6.0)	patients: 36/91 non-irradiated patients: 29/82	irradiated patients: 23/91 non-irradiated patients: 23/82		
Jadoul et al.(2017)	Belgium	retrospective longitudinal	8 (8 females)	leukemia mixed	no therapy details	NA	NA	NA	-	1997-2013	22.3 (SD ±8.8; range: 0.6–39)	~30	7.6 (SD ±3.5)	4/8	-	POI (self-reported in questionnaire)	-
Jensen et al (2016)	Denmark	retrospective cohort study	11 (11 females)	ALL; AML; CML	-	-	-	11	-	2000-2015	11.3 (range: 0.6–17.11)	„most patients“: 15–25	6	10/11	-	POI	-
Komori et al. (2019)	Japan	retrospective	11 (11 females)	ALL (4); AML (6); acute biphenotypic leukemia (1)	-	-	11	11 <8 GY	-	2003-2015	9.6 (range: 0.8-22.8)	19.7 (range: 13.4-26.7)	12.2 (range: 3.7-15.7)	3/11	-	POI	-
Letourneau et al. (2012)	USA	retrospective	60 (60 females)	mixed leukemia	60	-	-	-	-	1993-2007	28.5 (SD ±7.2)	36.8 (SD ±8.9)	8.3 (SD ±4.2)	12/60	-	WHO definition: ≥ 12 months no pregnancy conceived	-

Leung et al. (2000)	USA	retrospective longitudinal (St Judes)	77 (45 females; 32 males)	AML	group A: 44	group B: 18	group C: 15	group C: 15	10	1976-1989	group A: 9.5 (range: 0.2-20.1)<br>group B: 3.4 (range: 0.4-12.6)<br>group C: 8.4 (range: 0.4-17.3)	group A: 27.5 (range: 11.3-38.4)<br>group B: 21.9 (range: 14.8-29.2)<br>group C: 24.0 (range: 16.2-33.2)	16.7 (range: 10.1-23.5)	9/77<br>females: 6/45<br>males: 3/32	-	HRT	-
Maeda et al. (2003)	Japan	retrospective	27 (27 females)	CML (3); AML (12); ALL (12);	-	-	HSCT-TBI: 18;<br>HSCT+TBI: 9	9	-	1982-1997	11 (range: 5-16)	17 (range: 13-27)	5 (range: 3-14)	23/27	1/16	POI (FSH >25 or HRT)	FSH 10-25 IU/L
Mancini et al. (2008)	France	retrospective	14 (14 females)	mixed leukemia	NA	NA	NA	NA	-	~2002-2003	~36	38.1 (SD ±5.0)	~2	3/14	-	Self-reported infertility or postmenopausal state	-
Marquis et al. (2010)	Switzerland	retrospective longitudinal	11 (11 males)	ALL	11	-	-	-	-	~1990 - 2002	8 (range: 2-17)	22 (range: 19–29)	13 (range: 8–16)	0/11	3/11	azoospermia	FSH > 10 IU/L and/or oligospermia
Naessen et al. (2014)	Sweden	prospective longitudinal	22 (22 females)	ALL (6); AML (6); CML (10)	1	NA	21	NA	-	1998-2002	27<br>ALL: 21 (range: 15–33)<br>AML: 28	37	12 (range: 9–13)	21/22	-	amenorrhea	-

											(range: 15–43)<br><br>CML: 28 (range: 16-39)						
Nesr et al. (2024)	United Kingdom	retrospective longitudinal	11 (11 males)	11 CML	TKIs, no chemotherapy	-	-	-	-	2001 - 2020	34.4 (range: 22.9-45.4)	~39	5.1 (range: 2.5-16.5)	0/11	3/11	azoospermia	oligospermia
Netterlid et al. (2021)	Sweden	cross-sectional study	51 (51 females)	mixed leukemia	NA	NA	NA	NA	-	1964 - 2008	8.4 (range: 0.1-17.9)	34 (range: 19–57)	25 (range: 12–41)	8/51	-	POI	-
Poganitsch-Korhonen et al. (2017)	Finland	retrospective	17 (17 males)	ALL	non-alkylating agent: 10<br><br>alkylating agents: 7	+/- CI, number unknown	-	-	-	1979-1995	**age at diagnosis**: non alkylating: 5.3 (SD ±2.7; range: 1.1-11.3); alkylating: 7.3 (SD ±5.2; range: 1.7-16.1)<br>**age at biopsy**: non alkylating: 8.1 (SD ±2.8; range: 3.9-14.1); alkylating: 9.1 (SD ±4.3; range:	non alkylating: 22.9 (SD ±5.6; range: 18.0-32.0); alkylating: 22.6 (SD ±5.0; range: 18.0-29.0)	13.1 (SD ±6.7; range: 0.9–21.2)	2/17	-	azoospermia	-

											3.6-17.5)						
Romerius et al. (2010)	Sweden	retrospective longitudinal	21 (21 males)	mixed leukemia	chemo +/ irradiation	NA	-	-	-	1970–2002	9.6 (SD ±5.6)	30 (SD ±6.7)	19 (SD ±7.4)	4/21	-	azoospermia	-
Rosendahl et al. (2008)	Denmark	retrospective longitudinal	4 (4 females)	mixed leukemia	-	-	4		-	1999-2007	12	19.5	7.5	4/4	-	AMH <0.05 ng/ml	-
RoyMoulik el al. (2024)	India	retrospective longitudinal	42 (42 males)	CML	TKIs, no chemotherapy	-	-	-	-	2000-2022	10.8 (range: 1.1–19.1)	at least 18	7 (range: 0.25 - 21.75)	13/42	5/42	azoospermia	oligozoospermia or asthenozoospermia
Schmidt et al. (2005)	Denmark	retrospective longitudinal	5 (5 females)	AML (1); ALL (2); CML (2)	-	-	HSCT +/-TBI	+/- TBI, number unknown-	-	~1999 -2003	22.6	~25	2.17 (range: 1.5-3.75)	5/5	-	amenorrhea or HRT	-
Shimizu et al. (2012)	Japan	retrospective longitudinal	13 (13 females)	ALL (6); AML (5); CML (2)	0	-	13 MAC +/- TBI or RIC	8	-	1991 - 2010	14.9 (range: 12.2-18.1)	NA	at least 2 years	10/13 (9/10 MAC; 1/3 RIC)	-	amenorrhea	-
Wang et al. (2024)	China	retrospective	15 (15 females)	mixed leukemia	-	-	15	NA	-	2017 - 2023	22.8 (SD ±6.81)	NA	~2	15/15	-	amenorrhea	-
Wilhelmsson et al. (2014)	Sweden	retrospective longitudinal	17 (17 males)	AML ALL	conditioning busulfan-based or cyclophosphamide only	-	17	-	-	1978 - 2000	4.6 (range 1–17)	22 (SD ±6.0; range: 12–42)	13 (SD ±4.8; range: 4–28	17/19	-	azoospermia	-

Zaletel et al. (2004)	Slovenia	retrospective longitudinal	37 (37 females)	acute leukemia	37	6	0	1	-	1973-1992	5 (range: 1 -16)	19 (range: 13–31)	13 (range: 4–25)	1/37	-	amenorrhea	-

CI, cranial irradiation; TI, testicular irradiation; median or mean age at diagnosis, assessment and/or follow-up is given for the whole cohort of study participants, as it was rarely available for subgroups; range and standard deviation is given if available; gray shaded column of the table: fertility was assessed after cryopreservation on partial or entire ovary was performed for fertility preservation.

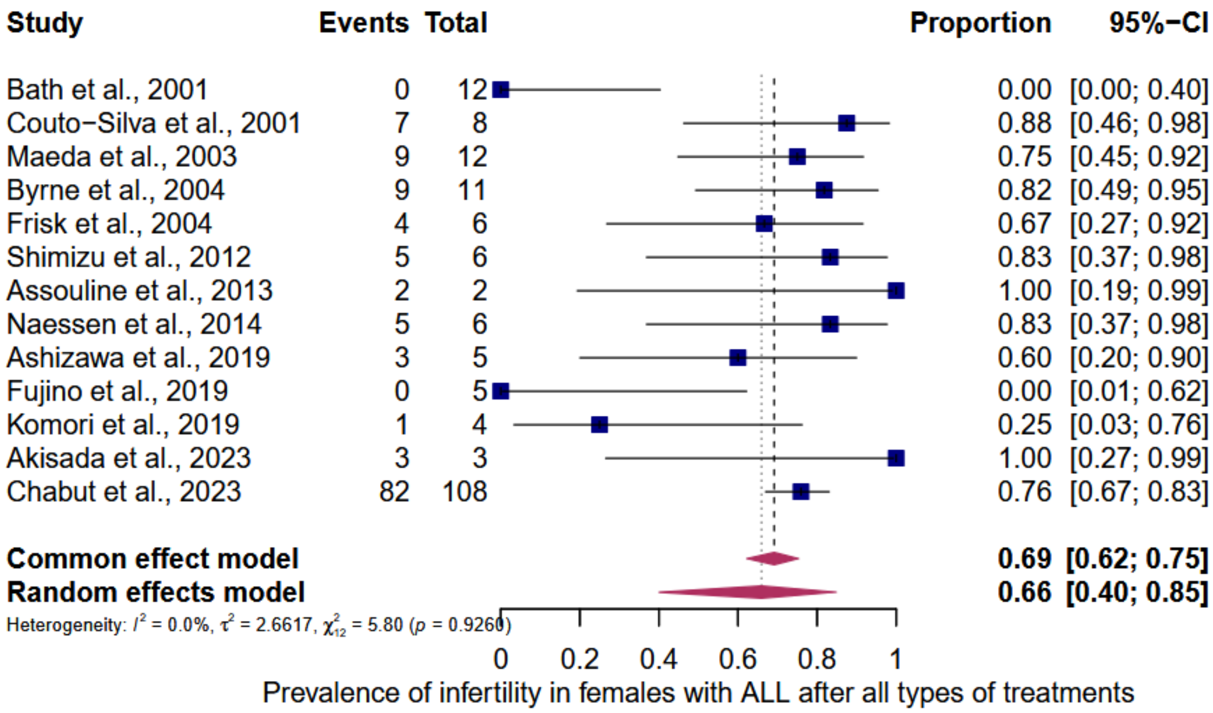

Figure S1. Prevalence of infertility in females with ALL.

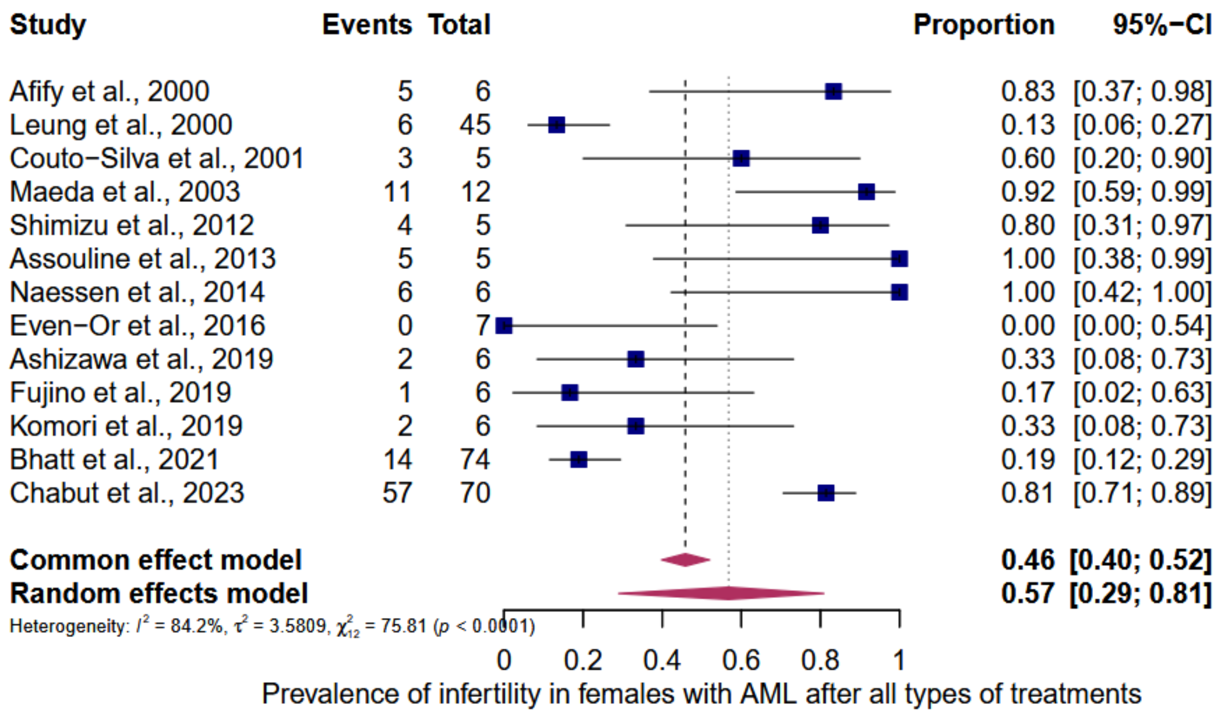

Figure S2. Prevalence of infertility in females with AML.

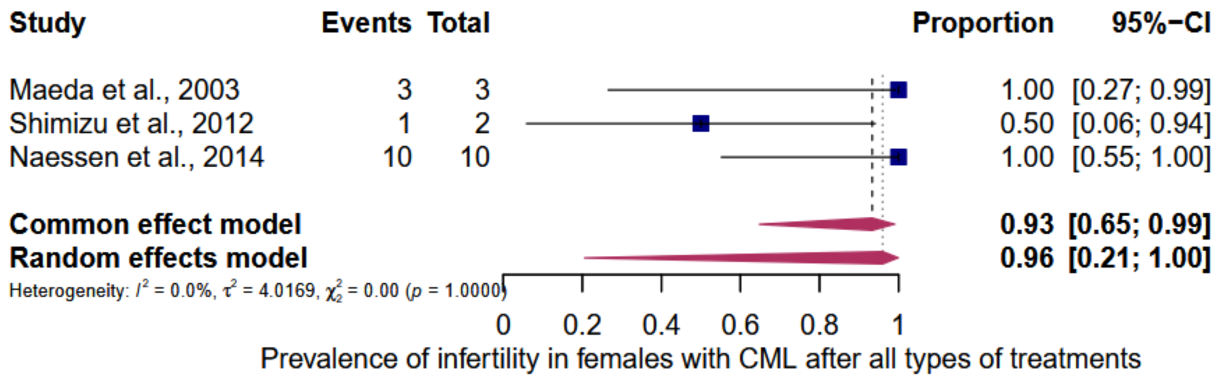

Figure S3. Prevalence of infertility in females with CML.

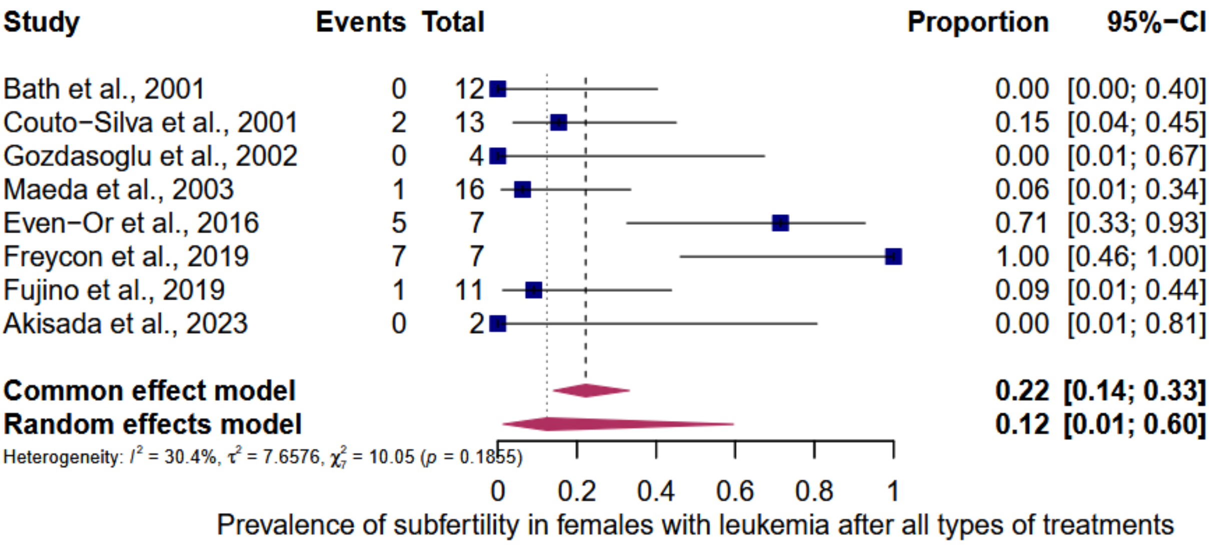

Figure S4. Prevalence of subfertility in females following all types of treatments.

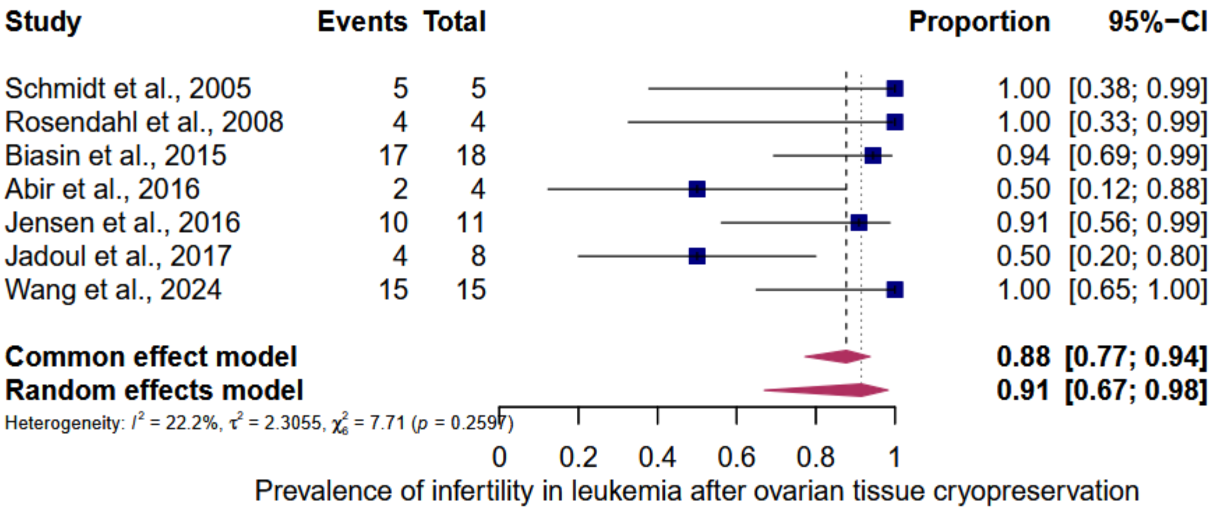

Figure S5. Post-treatment infertility in female leukemia patients after ovarian tissue cryopreservation.

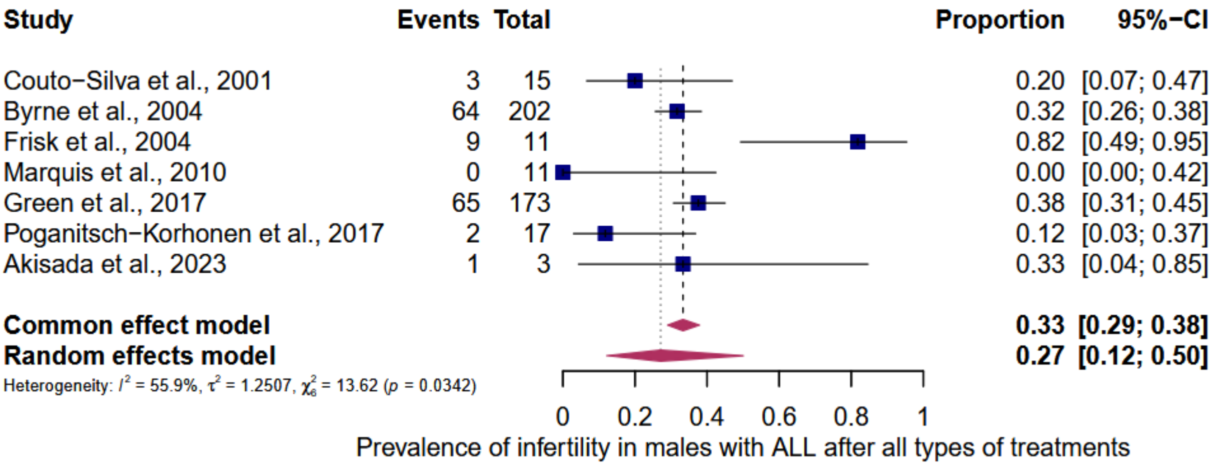

Figure S6. Prevalence of infertility in males with ALL.

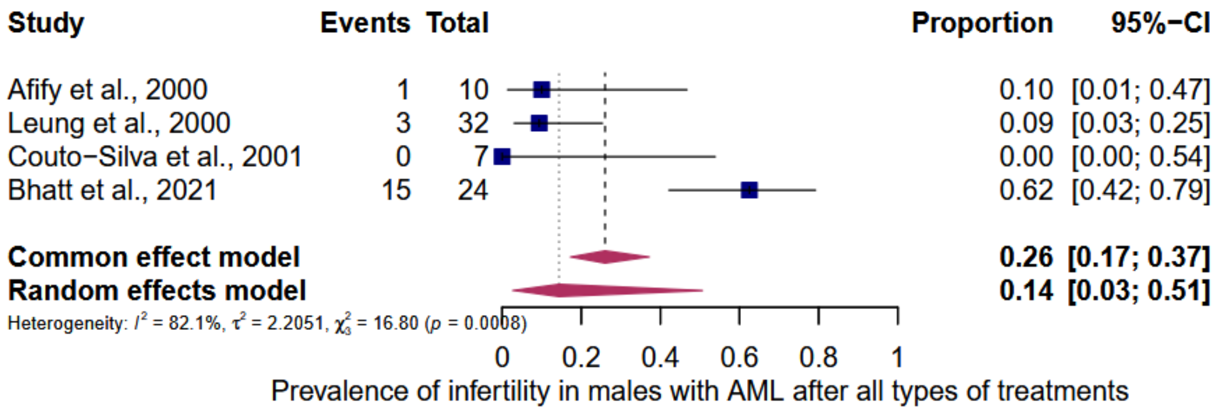

Figure S7. Prevalence of infertility in males with AML.

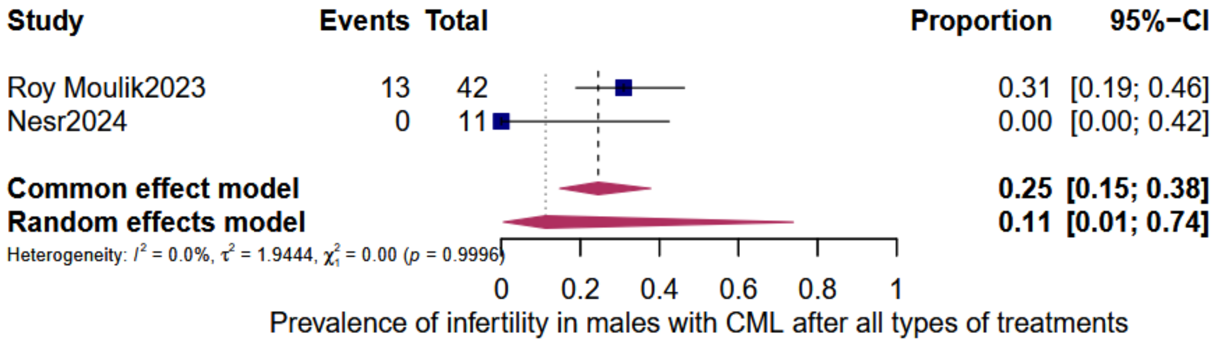

Figure S8. Prevalence of infertility in males with CML.

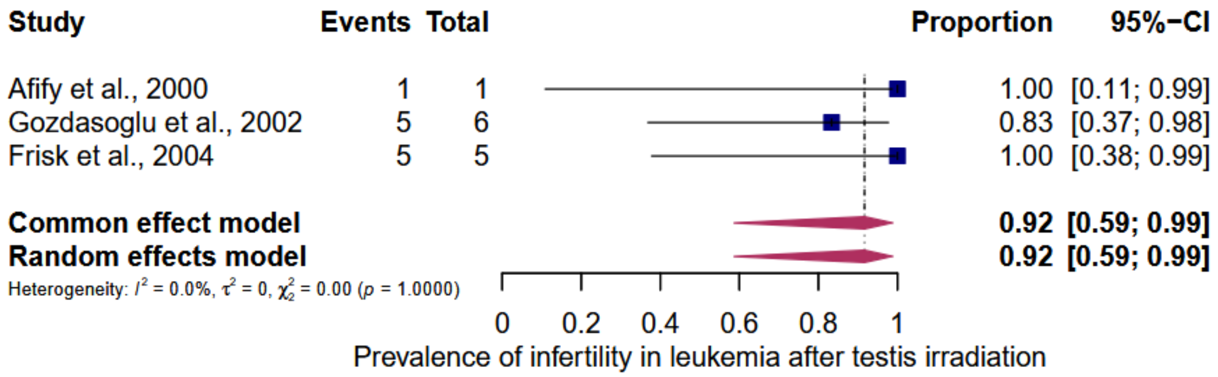

Figure S9. Prevalence of infertility in males after testicular irradiation.

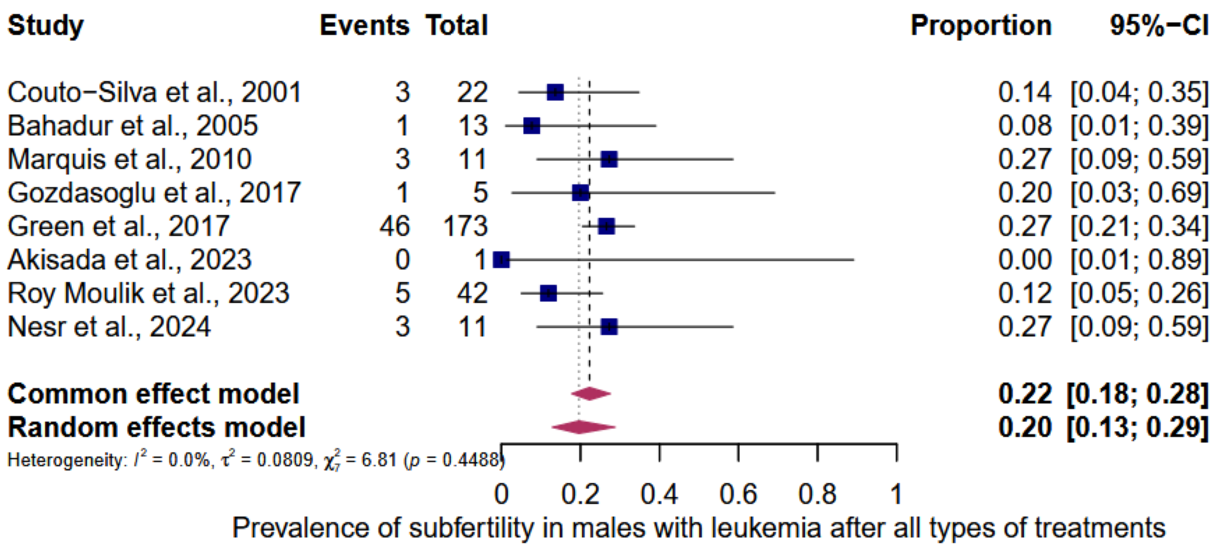

Figure S10. Prevalence of subfertility in males following all types of treatments.
